# A mixed methods feasibility evaluation of a shared decision-making intervention for community dwelling adults living with dementia, family carers and practitioners

**DOI:** 10.1136/bmjopen-2026-121790

**Published:** 2026-10-28

**Authors:** Jesutofunmi Aworinde, Clare Ellis-Smith, Nathan Davies, Elizabeth Sampson, Catherine Evans, On behalf of EMBED-Care, S Mead

**Affiliations:** 1 Cicely Saunders Institute of Palliative Care, Policy and Rehabilitation, King’s College London, London, UK; 2 National Ageing Research Institute Inc, Parkville, Victoria, Australia; 3 Centre for Psychiatry and Mental Health, Queen Mary University of London Wolfson Institute of Population Health, London, UK; 4 Academic Centre for Healthy Ageing, Whipps Cross Hospital, Barts Health NHS Trust, London, UK; 5 East London NHS Foundation Trust, London, UK; 6 Sussex Community NHS Foundation Trust, Brighton, UK

**Keywords:** Dementia, Health, Clinical Decision-Making, Family

## Abstract

**Abstract:**

**Objectives:**

To evaluate the feasibility of a shared decision-making intervention for people living with dementia at home, their family carers and healthcare practitioners.

**Design:**

A mixed-methods feasibility study and process evaluation using a multiple case study design at the individual level of persons with dementia, family carers and practitioners.

**Setting:**

The UK, National Health Service (NHS) community care (people living with dementia at home).

**Participants:**

People living with dementia at home, their family carers and NHS community healthcare practitioners who provide care to people living with dementia.

**Intervention:**

The intervention is the EMpowering Better End of life Dementia Care (EMBED-Care) Framework which we previously co-designed to enhance shared decision-making. The intervention comprises a holistic person-centred outcome measure (PCOM) known as the Integrated Palliative care Outcome Scale for Dementia (IPOS-Dem), linked with clinical decision support tools (evidence-based, simple flow diagrams of options to support decision making), priorities of care list (available in paper and digital formats), resources and training.

**Results:**

The study was made up of three cases, comprising a person living with dementia at home and/or their family carer and a practitioner. The EMBED-Care Framework has the potential to strengthen shared decision-making. Across three cases, joint use of IPOS-Dem indicated the potential to support deeper discussion of care and treatment and understanding the needs of people with dementia. The clinical decision support tools helped structure conversations about care and treatment options, prompting exploration of preferences with the person and/or family carer. The ‘priorities of care list’ was rarely used, indicating need for refinement and better integration into routine practice. Comprehensive staff training with ongoing and visible leadership support were identified as key factors for using the intervention.

**Conclusions:**

Using a holistic, PCOM alongside clinical decision support tools can strengthen shared decision-making. Together, they support collaboration between the person with dementia, family carers and practitioners, focusing discussion on needs and what matters most to the person and family. Future research will further explore implementation, undertake a pilot study to test the methods for a definitive trial and recruit a more diverse participant group.

**STRENGTHS AND LIMITATIONS OF THIS STUDY**

- This study used a case study design to enable an in-depth evaluation of the EMpowering Better End of life Dementia Care Framework for shared decision-making for people affected by dementia living at home, and practitioners.
- The cases in this study were at the individual level, which enabled an in-depth exploration of shared decision-making in different contexts, for example, dementia stage and ability of the person with dementia to be actively involved in decision-making.
- The study was underpinned by a theoretical shared decision-making framework which informed the development of the intervention (reported elsewhere) and its evaluation, reported in this study.
- Patient and public involvement and engagement was a key strength in this study that enabled the development as part of a large intervention aimed at improving palliative dementia care.
- A limitation of the study was the limited breadth of diversity of people with dementia with all identifying as White British and living with moderate dementia.

## Background

 Dementia is a life-limiting progressive illness that people die with or from. Globally, it is projected that dementia health related suffering will increase from 1.5 million in 2016 to 6 million by 2060.^1^ This means that the demand for care for people living with dementia in the community will increase. Globally, around 84% of people living with dementia live at home.^2^ People with dementia often rely on close family members to support them throughout the dementia trajectory. With the decline in cognitive capacity associated with dementia progression, increasing support and advocacy in decision making are required over time.^3 4^

Shared decision-making is necessary to align care provision with individuals’ preferences and priorities,^5^ with the intention to improve quality of life.^6^ Shared decision-making is a process that requires an exchange of information between practitioners, the person and their family.^7 8^ Shared decision-making ensures that the needs of people with dementia continue to be identified and addressed, despite the decline in cognitive capacity and verbal communication. Effective shared decision-making necessitates sufficient time and a high level of professional expertise. If poorly conducted, shared decision-making can be difficult for family carers to engage in, particularly if little support is offered by practitioners to engage in decision-making.^9 10^

There is evidence that person-centred outcome measures (PCOMs) can support communication and shared decision-making and improve outcomes in other populations, such as in patients with cancer.^11–15^ PCOM is a term sometimes used to describe measures that are designed and validated for self (patient) and proxy (eg, family carer) reporting of an individual’s own health status.^11^ Clinical decision support tools in palliative care have been shown to improve symptom management.^16^ These decision support tools can help clinicians provide more comprehensive care and prompt them to involve patients in decision-making.^16^ This highlights the value of using clinical decision support tools, particularly when they are linked to a PCOM. For example, a PCOM may highlight that a patient has pain, but a clinical decision support tool may help a clinician to consider potential causes of pain, particularly if the person cannot verbally communicate, but they have a family carer who can describe symptoms of the patient. Causes of pain may be dental or pressure sore. However, most of what is known about PCOM use in dementia is in care home settings, with little consideration of people with dementia living at home.^17–19^

In a previous study, we co-designed the EMpowering Better End of life Dementia Care (EMBED-Care) Framework for shared decision-making with people living with dementia and their family carers and healthcare practitioners.^20^ The intervention was designed for delivery by community nursing teams for people with dementia living at home. The intervention comprised a holistic PCOM to assess symptoms and concerns (the Integrated Palliative Care Outcome Scale for Dementia (IPOS-Dem)),^21 22^ linked with clinical decision support tools to manage care and treatment. The aim of this study was to evaluate the feasibility and the processes of the EMBED-Care Framework for shared decision-making for people with dementia living at home and their family carers and identify opportunities to strengthen the intervention. Using the results of this study, we refined an existing logic model of shared decision-making using the EMBED-Care Framework. The study objectives were: (1) To explore the processes of how (or not) the EMBED-Care Framework could enhance shared decision-making between people living with dementia (when able), family carers and healthcare practitioners. (2) To evaluate the feasibility (adherence and acceptability) of the EMBED-Care Framework to enhance shared decision-making (also referred to as ‘the intervention’ across this study). (3) Refine logic model from previous work that developed the intervention evaluated in this study.

## Methods

### Design

This mixed-methods feasibility study was registered at ISRCTN as part of the larger EMBED-Care programme (https://doi.org/10.1186/ISRCTN55008569). We evaluated the feasibility of the intervention, informed by the Medical Research Council Framework guidance on developing and evaluating complex interventions.^23^ We evaluated adherence, acceptability and capacity to deliver the intervention. The reporting was guided by the Consolidated Standards of Reporting Trials for pilot and feasibility studies^24^ and Consolidated criteria for Reporting Qualitative research checklist to enhance the structure, transparency and quality of reporting (online supplemental appendix 1).

We incorporated a process evaluation to understand *how* and *why* the intervention worked (or not) for people affected by dementia (including people with dementia and their family carers) at home and their practitioners within National Health Service (NHS) community team. The study explored the context in which the intervention was used and how this linked with mechanisms of impact (how participants interact with the shared decision-making intervention) and implementation factors (requirements to use the intervention in routine care)^25^ and its impact on outcomes.

The study design used a multiple case study informed by Yin.^26 27^ We used a case study design as it enabled understanding of the shared decision-making phenomenon in the real world context. Specifically, a case study design enabled in-depth exploration of multiple perspectives for each individual case. We also conducted cross-case analyses. A case unit of analysis was at the individual level (person with dementia). This is because shared decision-making requires patients to be the centre of care and treatment decisions. Each case included (1) A person living with dementia at home, (2) Their main family carer and (3) A practitioner who was a staff member in the participating NHS community nursing team, providing care for people living with dementia and using the intervention.

### Shared decision-making model

The Enriched Model of Collaborative Deliberation^7^ along with a logic model of how and why the EMBED-Care Framework could enhance shared decision-making, developed from our systematic review^5^ and co-design study, underpinned the understanding of shared decision-making in this study.^20^

### The EMBED-are Framework

The shared decision-making intervention was integrated within the EMBED-Care Framework, a larger intervention for use across community and care home settings, co-designed with people with dementia, family carers, practitioners and research over a series of workshops.^28^ Workshops were held specifically for the context of care at home. The intervention included:

1. A holistic PCOM (the IPOS-Dem) to identify symptoms and concerns^21 22^ (referred to as needs in this study). The IPOS-Dem is a PCOM, initially designed as a staff proxy measure for people with dementia living in care homes. It comprises multiple health domains, including physical, psychological and social well-being, and asks about family concerns.^21 22^
2. The IPOS-Dem items were each linked with evidence-informed clinical decision support tools to inform management of care and treatment and aid decision-making.^28^ Clinical decision support tools, known as ‘rules of thumb’ or ‘heuristics’, are methods for presenting the best available options to aid decision-making on care and treatment. They are displayed in simple flow diagrams to help consider possible options.
3. A priorities of care list was designed to identify and inform understanding on what mattered most to the person and/or family to prioritise and plan care and treatment.

The intervention package included face-to-face training for healthcare professionals, with resources such as short videos and intervention manuals made available. The Framework was available in both digital and paper versions.^28^ The digital technology company Aetonix were the creators of the ATouchAway platform that hosted the EMBED-Care Framework.^29^ The digital version of the intervention is an app that can be downloaded or used as a web-based platform. The digital component of the intervention has alert systems to prompt and remind users to complete the IPOS-Dem or to review clinical decision support tools. The platform had an in-built ‘dashboard’ for healthcare practitioners to monitor change over time and identify changes in symptoms and concerns, review outcomes of change in care and treatment.^28^ The intervention was implemented over a 3-month period. The shared decision-making intervention is illustrated in figure 1. This figure is from our co-design study^20^ and shows how the intervention works to enable shared decision-making. The shared decision-making element is informed by the Enriched Model of Collaborative Deliberation.^7^ A table with full intervention details, informed by the Template for Intervention Description and Replication Checklist,^30^ is reported in the online supplemental appendix 2.

**Figure 1:**
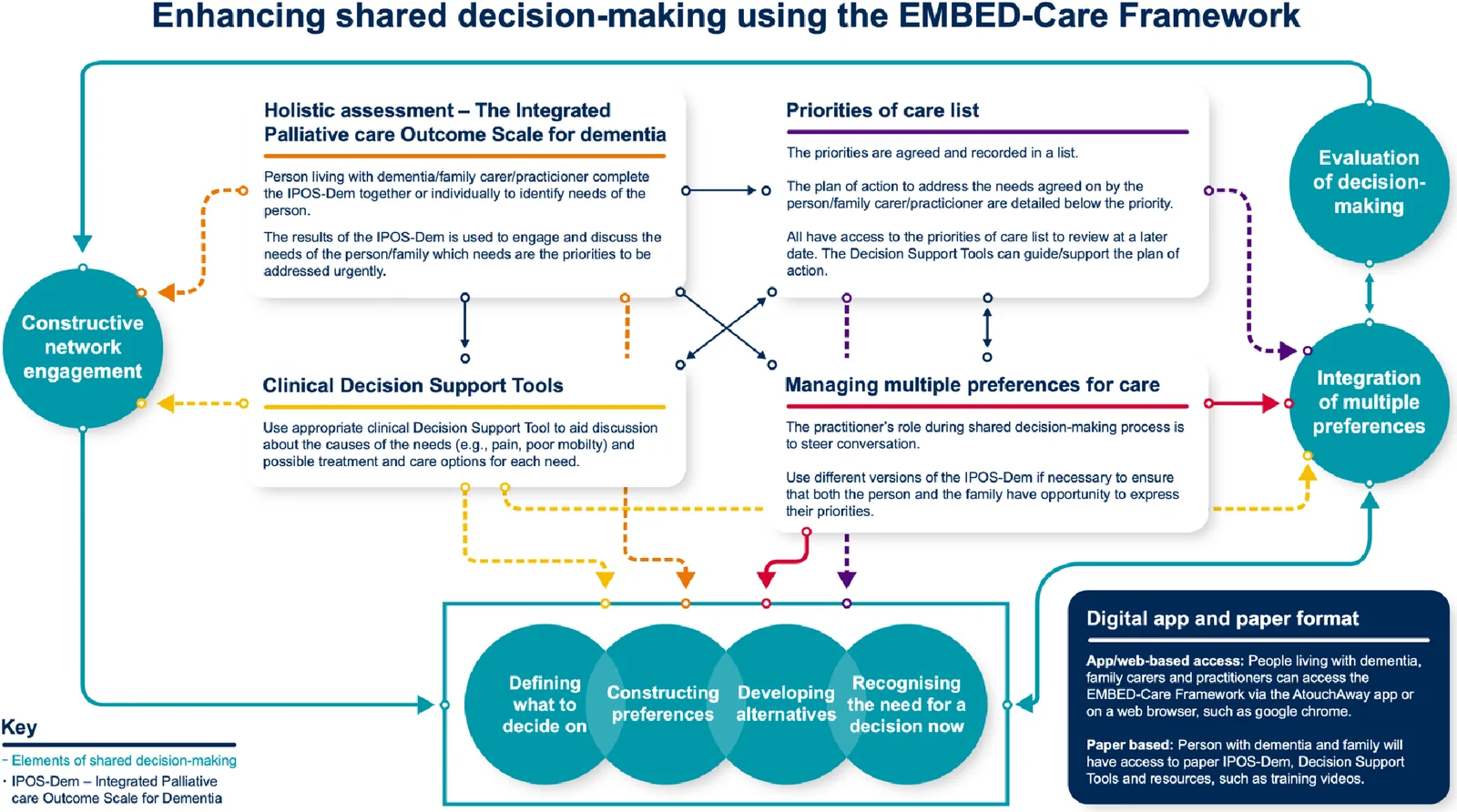
Intervention diagram, detailing how the individual intervention component interacts with different elements of shared decision-making. EMBED-Care, EMpowering Better End of life Dementia Care; IPOS-Dem, Integrated Palliative care Outcome Scale for Dementia.

### Patient, public involvement and engagement

A dedicated patient, public involvement and engagement (PPIE) group supported the study to ensure it remained relevant to shared decision-making for people affected by dementia and practitioners. The group comprised a person living with dementia, two family carers and a healthcare practitioner. The PPIE group met every 3–4 months from the study development stage until the end of the study and informed the development of the shared decision-making intervention and the feasibility evaluation study. The group then contributed to the development and refinement of the study topic guides for semi-structured interviews and focus groups.

### Study setting and participant recruitment

This study was conducted in an NHS community team. The NHS Trust covers both urban and rural areas, although the area is largely rural; care is delivered both in patients’ homes and in community hospitals. The community team served a population of around 1000 000 in 2022. People aged 60 years and above made up 33% of the population.^31^ 12% of older adults lived in low-income households. 93.7% of the Derbyshire population was of white ethnic origin.^31^ Prior to the study commencing, the research team developed a rapport with the clinical team to understand their processes. The clinical team was already using the IPOS-Dem as a core component of their routine care. However, using the IPOS-Dem together with people with dementia and family carers was a new aspect of the clinical team’s routine.

The number of cases within multiple case study designs vary. However, the recommended number of manageable cases is three to four varied cases.^32^ The intended sample was three cases as this was deemed sufficient due to the multiple sources of data collected. The cases were purposively sampled to ensure variation in the cases.^33 34^ The intention was to have a diverse set of cases that varied from each other to maximise exploration of the intervention within different contexts.

### Participant eligibility criteria

#### The person with dementia

- People living with dementia in their own home
- Diagnosis of dementia (any type, stage, at any age) or be suspected of having dementia
- Availability of an unpaid family carer who supports them
- Residing in their own home in the community (alone or with their family carer)
- With or without cognitive capacity to consent to participate in the study
- Availability of a personal or nominated consultee if the person with dementia does not have the cognitive capacity to consent

#### Family carer

- Current, unpaid family carer of an individual with a dementia diagnosis or suspected of having dementia and participating in the research study
- Has contact with the person with dementia at least every 2 weeks
- Able to read in English
- Able to consent to participation in the research study

#### Practitioner

- A practitioner within the participating community NHS team
- Provides care for a person with dementia in their own home and is participating in the research study with their family carer.

### Consent process

Participants were given at least 24 hours to read the participant information sheet and reflect. People living with dementia were provided an opportunity to discuss the study with their family to decide if they wished to participate. Any queries were answered by JA (researcher). For people living with dementia, the capacity to consent was assessed by the researcher, who had completed the Good Clinical Practice training.^35^

People with dementia were assessed for capacity to consent, guided by the Mental Capacity Act.^36^ If the person lacked capacity, a personal consultee (an unpaid individual who knew the person well, eg, a family carer) was approached. If family carers did not wish to act as the personal consultee, a nominated consultee (a paid practitioner) would be approached to complete the declaration form after reviewing the patient’s clinical case note and speaking to the practitioners who had frequent contact with the person with dementia.

### Qualitative data collection and analysis

Semistructured qualitative interviews were conducted remotely with participants over Microsoft Teams and recorded on a digital voice recorder by the lead author (JA) who was a male in the final year of his PhD Training fellowship and had over 6 years’ experience of data collection with people living with dementia and their family carers. A topic guide was developed, informed by the logic model from previous studies and using a shared decision-making model.^5 7 20^ The topic guide was used to explore how the participants used the intervention. Interviews were completed with people living with dementia (if they had capacity) together with their family carers. Practitioners were interviewed individually regarding their experiences.

The audio recordings (30 min on average) were transcribed verbatim by a transcription company. Transcripts were anonymised, using unique participant identifiers. Any description that risked unveiling the participants’ identities was anonymised to retain participant anonymity. The data were analysed using NVivo V.14. After familiarisation with the qualitative data, each transcript was coded, initially using a coding tree (online supplemental appendix 3) to organise the data in relation to shared decision-making. A case summary was produced for each case to evaluate the feasibility of the intervention and explore the processes of the shared decision-making intervention within each case. A narrative explanation was developed prior to data analysis for how and why the intervention was to work, informed by the logic model that provided the underpinning theoretical understanding of shared decision-making.^20^ This explanation was used in the cross-case data analysis where the data were analysed using reflexive thematic analysis.^37^ The explanation was compared with the results from each case during cross-case analysis. Yin’s ‘explanation-building’ technique^26 27^ was used to identify the patterns across all cases and themes generated on how the intervention was used for shared decision-making, and to understand the acceptability of the intervention.^27^ Thematic analysis was undertaken by the lead author. To ensure rigour, the data were iteratively coded. The lead author discussed and sense checked codes with coauthors before the themes were generated. Once the themes were generated, these were discussed with coauthors to ensure that the themes generated reflected the data.

### Quantitative data collection and analysis

Demographic data were collected, including age, gender and dementia severity (using the Clinical Dementia Rating (CDR) Scale),^38^ to describe the participants within and across cases. The CDR is rated on a scale from 0 to 3. A score of 0 indicates no dementia, 0.5 indicates ‘questionable’ dementia, 1 indicates mild dementia, 2 indicates moderate dementia and 3 indicates severe dementia. The CDR is a valid and reliable measure for assessing dementia severity.^38^

The Edmonton Symptom Assessment Scale (ESAS)^39 40^ was used as an indicator of the level of needs an individual had, based on the number of symptoms experienced. The ESAS has nine symptoms; each rated on a scale from 1 (no symptom) to 10 (worst symptom possible). For each symptom, a score of ≤3 is considered stable, 4–6 requires further assessment and ≥7 is unstable. We converted the number of symptoms that fall within each of these three categories into a score out of 100, using the ESAS guidance.

Monthly use of the intervention was generated by the digital technology company Aetonix.^29^ Quantitative data were analysed in Microsoft Excel, using grouping and counting techniques. Using the app data, the mean IPOS-Dem Score for each item at 4 weeks and 8 weeks was reported using a radar plot to illustrate adherence and item missingness (online supplemental appendix 4, case summaries). The baseline timepoint score was 0 as the intervention was not used at baseline.

### Integrating qualitative and quantitative data

The qualitative data were analysed first on a case-by-case basis. The quantitative data for each case was then analysed. Integration occurred at the analysis and interpretation stage. A joint display was used to merge and present the qualitative and quantitative data (online supplemental appendix 4) in the case summaries to highlight the similarities and differences across cases, guided by Haynes-Brown and Fetters.^41 42^ The findings of the study were used to refine an existing logic model developed in previous studies.^5 20^ The refined logic model details how and why the intervention works.

## Results

The study was conducted from February to April 2024 in the participating community NHS Trust. Twelve people (including people living with dementia (n=4) and their family carers (n=4)) were assessed for eligibility, and eight were recruited to form three cases (figure 2). Each case comprised a person with dementia (n=3), family carers (n=3) and practitioners (n=2). One practitioner was in two cases. The practitioners were nursing assistants, each reporting to a registered nurse. Of the participants with dementia, one had the capacity to give informed consent and provide self-report data. Two themes were generated from the qualitative data for how the intervention worked to enhance shared decision-making: (1) Theme 1: Establishing a relationship to build knowledge of the person and engage with the person’s network (including the family carer and healthcare practitioner). (2) Theme 2: Healthcare practitioner training and support to use the shared decision-making intervention.

**Figure 2:**
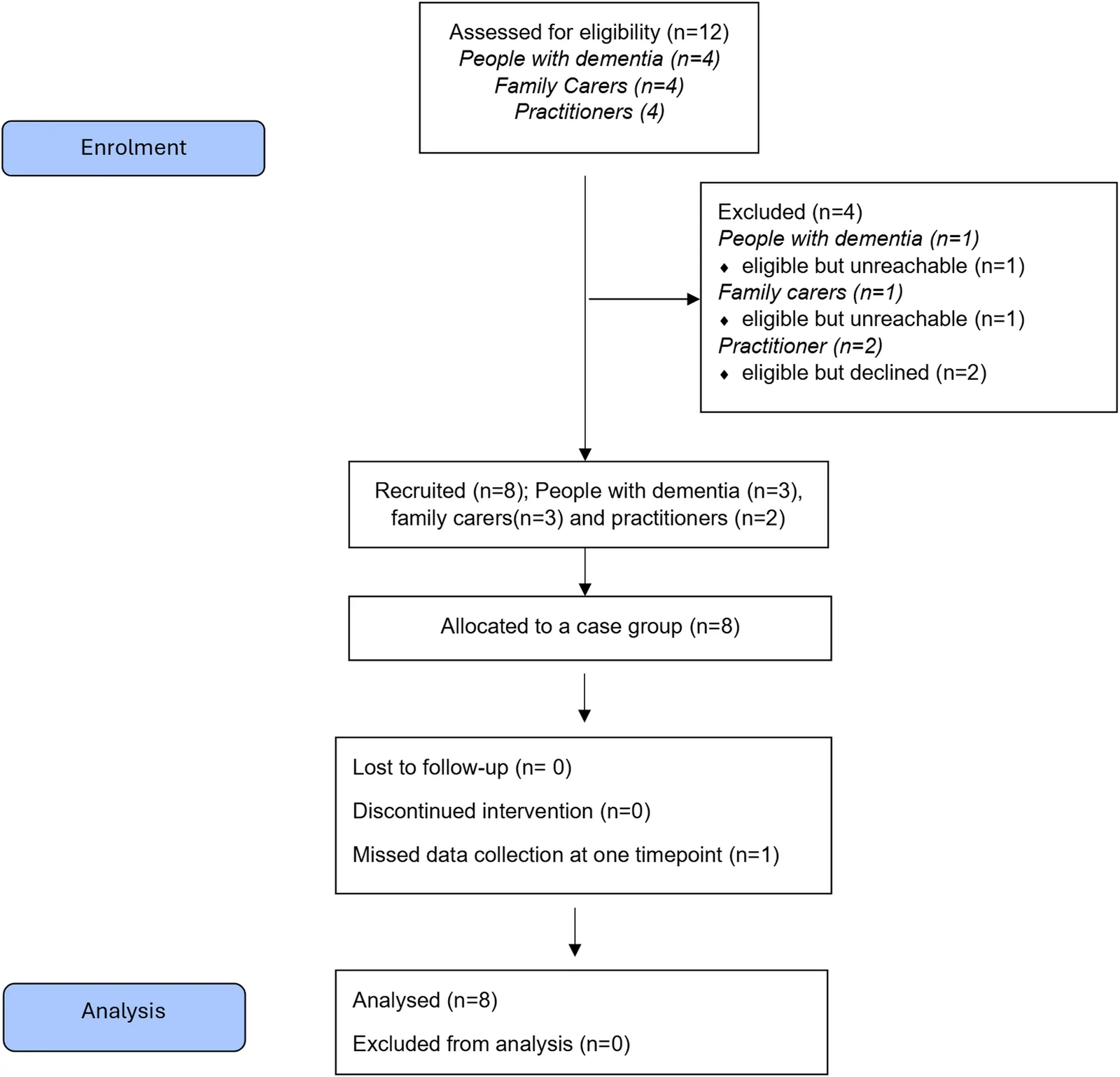
CONSORT flow diagram. CONSORT, Consolidated Standards of Reporting Trials.

### Participant characteristics

Table 1 details the characteristics of the respective participant groups. Two of the three participants with dementia had Alzheimer’s disease, and one had a rarer form of dementia (not specified to protect anonymity). Dementia severity ranged from mild to moderate with CDR^38^ ranging from 0.5 to 2 (questionable to moderate dementia). No participant had severe dementia. Two of the family carers were spouses of the person with dementia, and one was an adult child. Practitioners were two nursing assistants, one with over 10 years of experience working with people living with dementia.

**Table 1:** Participant characteristics

Participant demographic information			
	PwD (n=3)	Family carer (n=3)	Practitioner (n=2)
Age, median (IQR P25, P75)	75 (74, 78.5)	67 (63.5, 70)	
Gender (female), *n*	2	2	2
Dementia type, *n*			
Alzheimer’s disease	2		
Rare form of dementia	1		
Relationship between PwD and family carer			
Husband/wife	2		
Parent/child (family carer)	1		
Clinical Dementia Rating Scale category			
Mild dementia (score of 0–1)	2		
Moderate dementia (score of 2)	1		
Edmonton Symptom Assessment Scale, mean (SD)	41.33 (5.19)		
Practitioner role			
Nursing assistants*			2
Practitioner years of clinical experience			
<9 years			1
≥10 years			1

* All nursing assistants were paired with registered nurses.

PwD, person with dementia.

### Individual case summaries

The characteristics of people with dementia, family carers and practitioners are detailed in the individual case summaries that provide an in-depth context of how the intervention was used within each respective case. These are detailed in online supplemental appendix 4. For each case, the findings report baseline CDR and ESAS individual items. The total ESAS Score indicated low symptom severity, but individual symptom scores showed that participants had at least one symptom with high severity (score ≥5 out of 10), including breathlessness, fatigue, well-being, anxiety. Case 1 had five symptoms on the ESAS with a score of 5, while cases 2 and 3 had four each. High symptom severity (score of >5) is echoed in the intervention. In case 1, the IPOS-Dem Score showed that 7 and 15 symptoms, respectively, at 4-week and 8-week timepoints, affected the person with dementia either ‘moderately’, ‘severely’ or ‘overwhelmingly’. Case 2 reported 10 and 13 symptoms, respectively, over the same period. Case 3 reported 11 symptoms at the 4-week timepoint (online supplemental appendix 4, case 3 summary). Commonly reported symptoms included pain and weakness or lack of energy, drowsiness and family anxiety.

The findings on intervention acceptability and adherence are reported in each case by the respective objective, reporting both qualitative findings about acceptability and quantitative about intervention adherence (online supplemental appendix 4). In case 1, the intervention was used between the person with dementia, their family carer and the practitioner, who used a mix of digital and paper versions of the intervention. In cases 2 and 3, the family carers and practitioners used the digital version of the intervention.

### Cross-case process evaluation on whether and how the intervention could enhance shared decision-making

Across all three cases the participants used the digital version of the intervention together with a practitioner during a home visit. The IPOS-Dem indicated acceptability. Participants found the IPOS-Dem to be helpful in establishing rapport, and the involvement of family carers was required. When the IPOS-Dem was completed, there were no missing data across the cases. However, the clinical decision support tools were only used in cases 1 and 2, although infrequently.

#### Theme 1: Establishing a relationship to build knowledge of the person and engage with the person’s network (including the family carer and healthcare practitioner)

Across all cases, established relationships within each network were important for using the intervention. The rapport between the person and/or the family carer and practitioner put individuals at ease. The relationship between the participants across the cases allowed for ease of conversation. This was particularly important to the practitioners.

…because sometimes she [the family carer] does notice different symptoms, and we’re not going for another week or two, and she’ll be worried about them, or she won’t know what actions to take as such. Case 1 – practitioner

The intervention supported ‘knowing the person’ and understanding the needs of the person with dementia. The intervention component used during this stage of the shared decision-making process was the IPOS-Dem. The IPOS-Dem was used as a conversation starter in two cases to understand the symptoms being experienced by the person living with dementia. In one case, the person with dementia was involved in completing the IPOS-Dem, discussing symptoms and joint decision-making. In the other two cases, the individuals living with dementia could not be involved. To develop an understanding of the person’s needs, the person and/or the family carer were involved in the use of the IPOS-Dem.

It was, again it was fairly easy, I mean it’s how I see things basically rather than a clinician telling you what they saw, if you know what I mean, because the thing is that clinicians don't see every day all day type of things, and it’s quite useful actually. Case 2– family carer

The IPOS-Dem then encouraged reflection and collaboration with practitioners to think more deeply about the extent to which symptoms affected the person with dementia. This included needs that individuals may not otherwise have considered. The practitioner provided their perspectives on the symptoms, while those affected by dementia were able to share their experiences to contribute towards the discussion on the symptoms affecting them.

I thought it was very interesting and we did discuss some of the questions and some were asked… And it put everything into correct perspective. Case 1 – person with dementia

Across all cases, the intervention encouraged the person living with dementia and family carers towards ‘engaging and considering the perspectives of all involved in decision-making’ during the next stage of the shared decision-making process. The next step of the shared decision-making process using the EMBED-Care Framework was for the person living with dementia, family carers and healthcare practitioners to discuss the symptom priorities and care and treatment decisions. The intervention component that facilitated this stage of the shared decision-making process was the clinical decision support tools. The use of the decision support tools was present across all cases, led by the practitioners.

It weren't really until afterwards you're looking at the support tool and they made one or two notes of things to actually try and incorporate with what we do day-to-day, you know, when there’s no one here, it’s useful in that respect. Case 2– family carer

The use of the priorities of care list, developed for prioritising which symptoms to address, was limited to one use in only one case. The practitioners did not use this intervention component due to limited time during a home visit.

I've avoided that at the minute because I just wanted to do the IPOS[-Dem] with them, it was the first, first time I did it with both of them, so I'm going to have a look at it and [laughs] go with it from the next visit onwards, kind of thing. Case 1- practitioner

#### Theme 2: Healthcare practitioner training and support to use the shared decision-making intervention

Across all three cases, there was evidence to suggest the feasibility of the shared decision-making intervention. However, a major requirement for all participants was ‘time’ to become familiar with the intervention, specifically using IPOS-Dem, linking it with the clinical decision support tools. For the nursing assistants, the intervention took more time than anticipated at the first time of use. However, the time requirement decreased with experience and familiarity.

So that really did help and when you’ve done it a few times it kind of comes naturally, because it’s that easy to use when you’ve done it a few times. Case 3 – practitioner

While time was required to become familiar with the intervention, support from team leadership was necessary to encourage practitioners across the cases to use the intervention. The support was in the form of additional time during a patient visit.

So, if we know we're going to need extra time, we'll book extra time. So that’s one really good thing about our team, to be fair, we're quite flexible on that and we know that if it’s going to be sensitive conversations and things like that, we'll always book extra time. Case 1 – practitioner

Across all cases, participants reported the need for simplicity, both for practitioners and for people affected by dementia. Simplicity and brevity of the intervention increased the likelihood of use, particularly for the person with dementia. The easier it is to use the intervention, the better it is for comprehension.

But the boxes are really simple, and just easy, you know what you’ve got to click, and you know where everything is. Rather than being, you know, scrolling down the whole list of things, I think the layout of the app really helps the simpleness of it. Case 3 – practitioner

Practitioners across all three cases welcomed the comprehensive training provided along with resources, supporting them in using the intervention. The practical component of the training to use the app was valued as it enabled practice and familiarisation.

Yeah, very much so, yeah, because you can practise on it as much as you like kind of thing and you know it doesn't matter if you press the wrong button or put something, it doesn't matter. Case 2 – practitioner

Lastly, flexibility in how the intervention could be used was important for participants across the cases. For example, completing the IPOS-Dem together or prior to a practitioner home visit, or using the paper version of the intervention rather than the digital version, enabled individuals to increase the likelihood of engaging in shared decision-making.

Yeah, [the practitioner] should come round… Because we can, any points, we can raise them straightaway and be clear, clear about what they're really getting at and want to know. Case 1 – person with dementia

### Feasibility (adherence and acceptability) of the shared decision-making interventions

In two cases, the intervention was used at timepoint 1 (4 weeks after baseline) to assess needs, and at timepoint 2 (8 weeks after baseline) to review and monitor care. In one case, the intervention was used once over the 2-month intervention period. Across all three cases, family carers were involved in using the intervention. However, there was only one case in which the person with dementia could be involved. The people with dementia in two cases could not participate as one was non-verbal and the other was unwell and hospitalised during the study. The intervention as a whole was perceived to be ‘helpful’, ‘easy to use’ and ‘straightforward’. Use of the intervention is further broken down by components in online supplemental appendix 4.

The intervention was used at least monthly, or sooner if clinically required. When the IPOS-Dem was completed, 100% of the IPOS-Dem questions were answered, and the ‘cannot assess’ option was not used, indicating that the IPOS-Dem was feasible as a holistic assessment, particularly in understanding the person’s needs. Participants perceived the IPOS-Dem positively.

So the actual form, like wandering as a result of distress or putting a person at risk, that gives more insight into what they're talking about with regard to wandering. So that sort of little comment in brackets on the form is extremely useful… Case 1 – family carer

The clinical decision support tools indicated the potential to support shared decision-making processes. In two of three cases, the clinical decision support tools were used with the person and/or their family carer to inform care and treatment decisions each time after the IPOS-Dem was completed. Although the clinical decision support tool was perceived to be helpful in one case, the practitioner felt that the information from the clinical decision support tools was overwhelming.

I think the biggest thing is… if you've got more than one symptom it kind of throws a lot of information back, and it’ s picking up, it’s almost skimming out what you don't need a little bit. Case 1 – practitioner

The findings indicated that the priorities of care list was used infrequently, suggesting that it was not feasible in its current format to support shared decision-making processes. The practitioners expressed the lack of time to use this intervention component and concerns regarding duplication of work.

I think at that point it was probably time, because I'd already been in the visits, both visits quite a long time, and I know we're quite good with flexibility with time, but I'm just wary that I'd got other visits booked and it had already taken quite a long time to go over everything and explain it thoroughly so they knew what we were doing. Case 1 – practitioner

## Discussion

This study took a novel approach by applying a multiple case study design to evaluate the feasibility and processes of the shared decision-making intervention. The findings from this study indicate that some elements of the intervention could enhance shared decision-making. These were the IPOS-Dem and clinical decision support tools. The priorities of care list requires further revision to increase the likelihood of use.

The initial logic model, derived from the findings of our systematic review^5^ and subsequently refined through the co-design study,^20^ was further revised in light of the results from the present study (figure 3). The logic model displays how the intervention for people affected by dementia at home links to mechanisms of impact and outcomes. For example, the IPOS-Dem is important for bringing together a person’s network to develop an understanding of the person’s symptoms and concerns, which in turn links with outcomes relating to communication for family carers. The main findings are depicted in orange text in figure 3. For example, the IPOS-Dem and clinical decision support tools were key components of the intervention that were used; however, the priorities of care list was not. These intervention components, designed for use digitally and in paper form, linked with mechanisms of impact and shared decision-making, such as ‘knowing the person’ through using the IPOS-Dem. Whereas, ‘engaging and considering the perspectives of the person and family carer to determine priorities for care of all involved in decision-making’ was managed using the clinical decision support tools. The logic model also shows gaps in understanding which interventions require further exploration, such as more detailed health outcomes for people with dementia.

**Figure 3:**
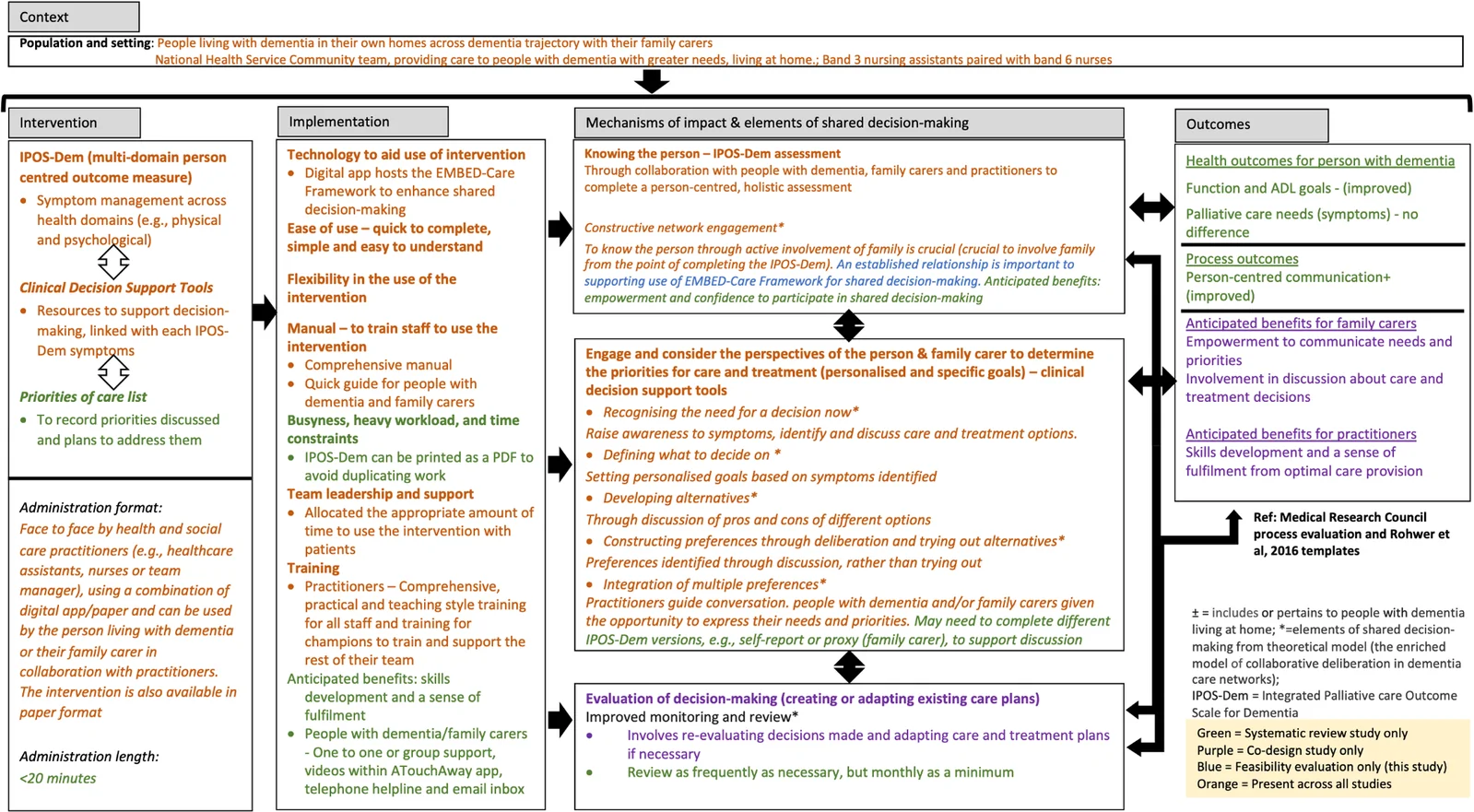
Logic model of the shared decision-making intervention, incorporating the EMBED-Care Framework. EMBED-Care, EMpowering Better End of life Dementia Care; IPOS-Dem, Integrated Palliative care Outcome Scale for Dementia.

A crucial finding of using the intervention was knowing the person and understanding the symptoms and concerns affecting the person with dementia. This required the involvement of the person and/or their family carer in the completion of the IPOS-Dem. Important to the shared decision-making process was the necessity for an established rapport within the person’s network, which included the person themselves or their family carer and a practitioner.^7^ This aligned with the underpinning theory of shared decision-making.^7^ Second, engaging the perspectives of people affected by dementia was key to determining the priorities and preferences for care and treatment. The intervention component that enabled this was the clinical decision support tools. This finding aligns with existing evidence on the benefits of clinical decision support tools when multiple decisions need to be made.^43 44^

An important gap in the evidence on using the EMBED-Care Framework for shared decision-making was related to managing multiple preferences for care and treatment.^20^ In this study, there were no opportunities to evaluate this, possibly due to the small sample size and limited diversity in the sample. The existing evidence on successful shared decision-making in advance care planning highlights the importance of family carers having the opportunity to be involved by sharing their knowledge and experiences and preferences for care.^45^

Training for practitioners that included a practical element was important for understanding and using the intervention. A practice account was provided to practitioners to increase their confidence in using the intervention, therefore signalling that the training was feasible. Existing evidence shows that training increases confidence in the use of interventions in clinical care.^46–48^

### Strengths and limitations

This study has some strengths, including evaluation of the intervention on an individual level, enabled by use of case studies. Prioritising the individual level enabled a more focused exploration of how shared decision-making unfolds in practice and how it can be supported through the intervention. Second, virtual data collection, both for quantitative and qualitative data, permitted a rich data set, which may otherwise have been difficult to obtain due to the location of the recruitment site.

Our study sample was small due to the use of a case study design. Case study designs in feasibility evaluations are uncommon for evaluating the intervention feasibility and are often used at the organisation or site level. However, a case study design was chosen as it allowed for exploration of shared decision-making at the individual level which a traditional feasibility study design does not allow. Shared decision-making, using the intervention, could also be explored from the perspectives of the person with dementia, family carers and healthcare professionals. These multiple perspectives made the study unique and provided more in-depth understanding of shared decision-making in the context of people living with dementia in their own homes.

This study encountered recruitment challenges which affected the sampling. We intended to recruit from two different community teams in the UK. However, there were delays in confirming capacity and capability to undertake the research in the second NHS community location. This compressed the study timeline, and a pragmatic decision was made to proceed with one NHS community team. This change in the original study plan also affected the recruitment plan. The second challenge was the opening time of the second study site. The delays in opening compressed recruitment time. It was therefore not possible to sample the cases as originally planned. The planned sampling characteristics included individuals from ethnically diverse backgrounds, dementia severity using the CDR Scale to identify participant severity ranging from mild to severe dementia, and level of clinical needs (using the ESAS) ranging from little to severe clinical needs. Although participant characteristics were described, the planned sample was not possible due to the limited timeline as a result of recruitment challenges. An exploration of the intervention with people from diverse backgrounds would provide a comprehensive understanding of how the shared decision-making intervention could work across different cultures and backgrounds and be more widely applicable for use with all. An important consideration is to recruit from locations with more ethnic diversity.

## Conclusion

The results showed that some aspects of the intervention could enhance the shared decision-making process. The IPOS-Dem was helpful in identifying and managing holistic needs for people living with dementia. IPOS-Dem facilitated conversations between people living with dementia and/or their family carers and nursing assistants through shared completion of the IPOS-Dem between the person and/or their family carer, and practitioner, to identify symptoms and concerns of the person and family carer over the past week. Once the IPOS-Dem was completed, clinical decision support tools, linked with symptoms on the IPOS, enabled discussion between practitioners and people living with dementia and/or their family carers regarding exploration of preferences for care and treatment. This decision was a collaborative effort. The results indicate that the intervention, particularly the IPOS-Dem could be used by nursing assistants who could report to registered nurses, thus fostering a thorough care process. However, revision of the other intervention components is required to strengthen feasibility, such as revised clinical decision support tools to make them quicker to use. The intervention was largely perceived to be useful and helpful in making and implementing shared decisions. Future research will include more individuals living with dementia who are able to be actively involved in the shared decision-making process. Future research will also explore implementation, undertake a pilot study to test the method for a definitive trial and recruit a more diverse participant group.

## Supplementary material

10.1136/bmjopen-2026-121790 online supplemental file 1

## Data Availability

Data are available on reasonable request.
