# Supplementary material for: A mixed methods feasibility evaluation of a shared decision-making intervention for community dwelling adults living with dementia, family carers and practitioners
Source: BMJ Open. 2026 Oct 28;16(10):e121790. doi: 10.1136/bmjopen-2026-121790 (PMC13723839; doi:10.1136/bmjopen-2026-121790)
Supplement: Supplementary file 1 [file bmjopen-2026-121790.reviewer_comments.pdf]

PEER REVIEW HISTORY

BMJ Open publishes all reviews undertaken for accepted manuscripts. Reviewers are asked to complete a checklist review form and are provided with free text boxes to elaborate on their assessment. These free text comments are reproduced below.

ARTICLE DETAILS

Title (Provisional)

A mixed-methods feasibility evaluation of a shared decision-making intervention for community dwelling adults living with dementia, family carers and practitioners

Authors

Aworinde, Jesutofunmi; Ellis-Smith, Clare; Davies, Nathan; Sampson, Liz; Evans, Catherine; ., On behalf of EMBED-Care

---

VERSION 1 - REVIEW

**Reviewer**	**1**
**Name**	**Jones, Steven**
**Affiliation**	**University of Chester, Public health school**
**Date**	**16-Jul-2026**
**COI**	**None**

A feasibility assessment is a comprehensive evaluation used to determine if a proposed project is viable before committing significant time and resources.

It identifies potential risks, obstacles, and the likelihood of success to reduce failures.

Study-

Clear background section in a neglected area of research.

Some minor syntax issues in methods section, from second paragraph.

P9 - PPIE- did the study engage PPI input at early stage of the project , at planning stage for example?

Study AIMS/ OBJECTIVEs (and if any research question) need ;prominence / clarity near methods . I noted them in abstract only.

P9 - Study setting , did the group consider gender and ethnicity factors that might arise and how they might be addressed. Accepting the demographic for the area ?

P10. Qualitative data analysed manually from transcripts which is acceptable, but make that clear. How was quality of thematic analysis (process) maintained or any disputes resolved? Plus why software package not used such as NVIVO etc. Qualitative phased approach to

reflexive thematic analysis is a systematic way to evaluate the results. Just needs some clarity on this , a few lines will suffice.

Accepting that multiple case study design is suitable to understand shared phenomenon in its real world context. Might be good to have sentence in the methods section (to inform readers that might not be familiar with it).

p21- Strengths and limitations. Accepting small study sample size. Need a few more lines on why case study design was chosen, what strengths it ' brought to the project over other designs' . It is mentioned in second para but needs defending / emphasis. Especially as case study designs are not used more generally within feasibility studies.

Would like to read more on the ' recruitment challenges encountered ' and how this might have been anticipated/ mitigated. I am surprised by this. What might be done next time for other researchers in this area?

**Reviewer**	**2**
**Name**	**Gamble, Andrew**
**Affiliation**	**The University of Sydney**
**Date**	**18-Jul-2026**
**COI**	**None**

I thank the authors for the opportunity to review this paper on the feasibility evaluation of a shared decision-making intervention for community dwelling adults living with dementia, family carers and practitioners. Overall, it was difficult to follow this study's aim and methods. I have left some more details comments below.

Abstract:

Setting – more detail is needed here – Country? State? It says community care, but the title suggests it is people with dementia living at home? Please clarify

It is not clear if the EMBED-Care is a pre-existing framework – I suggest stating 'we previously developed…' if it was developed by the authors for feasibility evaluation. (I see this is mentioned in the strengths and limitations but it is not clear in the abstract)

19 – participants could be more specific (as per strengths and limitations)

31 – potential of the EMBED-Care framework to support SDM using the IPOS-Dem? Please be specific to clarify is this outcome part of the framework or is the outcome measure used to measure feasibility of the intervention?

33- what are the clinical decision support tools in the framework? is not clear.

44 – Conclusion - The conclusion focuses on the outcome measure and support tools from the framework, but it is not clear what was used to determined feasibility of the intervention

on shared decision-making and why it only mentioned some aspects of the framework? It is not clear what the framework includes and what the tools are. This could be described in more detail in the intervention, if possible, with word count limits.

Page 5

Background

8 – people suffering?

40 – can you give an example of a shared decision-making tool?

45- as per my comment above, I suggest clear terminology between in-home resident of community care/living at home to avoid confusion.

Page 6

5 – this seems like a result that should not follow the aims in the background section. I suggest ending with the aim or describing the logic model above with an explanation that is also an aim to refine it.

Methods

Design – these measures were not mentioned in the abstract and there could be greater focus on how feasibility of the intervention is evaluated.

Page 7

- The text size seems inconsistent

5 – this could be more concise. It could be more specific if it is talking about the co-design process of developing the framework or the intervention itself. Sub heading may help to describe each of the components of the intervention and what it involves.

7 – see comment above for clarifying setting.

Figure 1 – I like the visual representation, but this is difficult to follow. Maybe some numbers or subheadings could help direct more intentional order to what should be viewed first/flow of the visual that can be followed or make it clear what the arrows mean? It may need to be simplified into the elements of the intervention separated from the elements of shared decision-making?

Page 9

- Please check text size throughout

- Try to avoid crossing between the previous study and this study too much. Unless I missed it, this is the first mention of interviews? Are they part of this study? It would be helpful to include this in the abstract or definition of how feasibility was planned to be evaluated in the abstract methods. Or results of how many were conducted and with whom

Setting – in the United Kingdom? A community team helping people with dementia living in their home?

Results

Page 11

- Please be more specific – 12 people? Is this everyone or people with dementia etc

Page 14

- I suggest describing a summary of table 11 then just have '(Table 11)' should this be table 1??

- There needs to be a description of the scales used and scoring for each and definitions of Alsheimer's etc

Page 17

- A summary of text should be provided describing the themes and subthemes before they are presented. As mentioned above it did not seem clear that interviews and themes were part of this study.

- This section is too long and should be more concise. I suggest using an appendix.

Discussion

Page 20

8 – ". The findings from this study indicate that intervention could enhance some elements of the intervention.' – this does not make sense? Two elements?

18 – perhaps a summary of what change in the logic model would be more helpful?

Conclusion

- As above, for someone not familiar with the framework, each element should be described for better understanding with a clear focus on the evaluation of the feasibility across three case studies of the framework. For example, it would be helpful to describe how the outcome measure in the framework is intended to facilitate shared decision-making. E.g. who delivers the outcome measure in the framework, how was it delivered? What does it consist of? And how does it facilitate shared decision making? And then a conclusion of how this was shown to be feasible e.g. interviews with who? suggested what? This could be expanding on the sentence "The IPOS-Dem facilitated conversations between people living with dementia and/or their family carers and nursing assistants" but while an outcome may identify needs how does it lead to shared-decision making? And how did it facilitate conversations? Between who? And were conversations management decisions? Or did this mean patient values and preferences were identified? Was this from interview findings? Observations?

VERSION 1 - AUTHOR RESPONSE

Reviewer 1 comments			
**4**	A feasibility assessment is a comprehensive evaluation used to determine if a proposed project is viable before committing significant time and resources.<br>It identifies potential risks, obstacles, and the likelihood of success to reduce failures.<br>Study-<br>Clear background section in a neglected area of research.	Thank you for your comment on the importance of this topic area and our decision to undertake a feasibility study. We feel this is an important topic area, but also appropriate research method to evaluate the topic.	
**5**	Some minor syntax issues in methods section, from second paragraph.	Thank you for highlighting the syntax errors. We have corrected these across the methods section.	Pages 5-12.
**6**	P9 - PPIE- did the study engage PPI input at early stage of the project, at planning stage for example?	Thank you for highlighting this point. We have updated this section to specify that the PPIE group was engaged from the study inception until the end.	"The PPIE group met every three to four months from study development stage until the end of the study..."<br><br>Page 9, lines 4-5.
**7**	Study AIMS/ OBJECTIVEs (and if any research question) need ;prominence / clarity near methods . I noted them in abstract only.	Thank you for asking for clarity regarding the study aim and objective. The study aim is detailed on pages 4 and 5. We have added our objectives. This is detailed on page 5.	"The study objectives were: (1) To explore the processes of how (or not) the EMBED-Care Framework could enhance shared decision-making between people living with dementia (when able), family carers and healthcare practitioners. (2) To evaluate the feasibility (adherence and acceptability) of the EMBED-Care Framework to enhance shared decision-making (also referred to as 'the intervention' across this study. (3) Refine logic model from previous work that developed the intervention evaluated in this study."<br><br>Page 5, lines 6-12.
**8**	P9 - Study setting, did the group consider gender and ethnicity factors that might arise and how they might be	Thank you for highlighting this important point regarding sampling characteristics. We did	"This study encountered recruitment challenges which affected the sampling. We intended that we intended to

	addressed. Accepting the demographic for the area ?	consider gender and ethnicity. We used purposive sampling with the intention of maximising variation in our sample, including gender and ethnicity. We also intended to recruit from two research sites, however, due to recruitment challenges and limited timelines, we were unable to achieve the diversity of gender and ethnicity we intended. We detailed this in the strength and limitations of the manuscript.	recruit from two different community teams in the UK. However, delays with confirming capacity and capability to undertake the research study in the second NHS community location. This compressed the study timeline and pragmatic decision was made to proceed with one NHS community team. This change in the original study plan also affected the recruitment plan. The second challenge was the opening time of the second study site which. The delays in opening compressed recruitment time. It was therefore not possible to sample the cases as originally planned. The planned sampling characteristics included individuals from ethnically diverse backgrounds, dementia severity using the Clinical Dementia Rating scale to identify participant severity, ranging from mild to severe dementia, and level of clinical needs (using the ESAS), ranging from little to severe clinical needs. Although participant characteristics were described, the planned sample was impossible due to the limited timeline caused by recruitment challenges. An exploration of the intervention with people from diverse backgrounds would provide a comprehensive understanding of how the shared decision-making intervention could work across different cultures and backgrounds and more widely applicable for use with all. An important consideration is to recruit from locations with more ethnic diversity."<br><br>Pages 21, lines 17-33 & page 22, lines 1-5.
**9**	P10. Qualitative data analysed manually from transcripts which is acceptable, but make that clear. How was quality of thematic analysis (process) maintained or any disputes	Thank you for raising this point. We did use Nvivo for analysis. We have added this detail to the methods section.	"The data was analysed using Nvivo 14."<br><br>Page 10, line 32, and page 11, line 1.

	resolved? Plus why software package not used such as NVIVO etc. Qualitative phased approach to reflexive thematic analysis is a systematic way to evaluate the results. Just needs some clarity on this, a few lines will suffice.	We have also added information regarding the thematic analysis process for clarity of the data analysis process.	"Thematic analysis was undertaken by the lead author. To ensure rigour, the data was iteratively coded. The lead author discussed and sense checked codes with co-authors before themes were generated. Once themes were generated, these were discussed with co-authors to ensure that the themes generated reflected the data."<br><br>Page 11, lines 11-14.
**10**	Accepting that multiple case study design is suitable to understand shared phenomenon in its real world context. Might be good to have sentence in the methods section (to inform readers that might not be familiar with it).	Thank you for raising this important point. We have updated this section to reflect the reviewer's suggestion.	"We used a case study design as it enabled understanding on shared decision-making phenomenon in real world context. Specifically, a case study design enabled in-depth exploration from multiple perspectives for each individual case."<br><br>Page 5, lines 28-30.
**11**	p21- Strengths and limitations. Accepting small study sample size. Need a few more lines on why case study design was chosen, what strengths it ' brought to the project over other designs'. It is mentioned in second para but needs defending / emphasis. Especially as case study designs are not used more generally within feasibility studies.	Thank you for raising this point. Further justification has been provided on the benefits of case study design, particularly in this study. We have also added an explanation on why case study was chosen.	"However, case study design was chosen as it allowed for exploration of shared decision-making at the individual level which a traditional feasibility study design does not allow for. Shared decision-making, using the intervention could be explored also from the perspective of the person with dementia, family carers and healthcare professionals. These multiple perspectives made the study unique and provided more in depth understanding of shared decision-making using in the context of people living with dementia in their own homes."<br><br>Page 21, lines 17-22.
	Would like to read more on the ' recruitment challenges encountered 'and how this might have been anticipated/ mitigated. I am surprised by this. What might be done next time for other researchers in this area?	Thank you for requesting more information on the challenges faced. We have added this detail to the strengths and limitation.	"This study encountered recruitment challenges which affected the sampling. We intended that we intended to recruit from two different community teams in the UK. However, delays with confirming capacity and capability to undertake the research study in the second NHS community

			location. This compressed the study timeline, and a pragmatic decision was made to proceed with one NHS community team. This change in the original study plan also affected the recruitment plan. The second challenge was the opening time of the second study site which. The delays in opening compressed recruitment time. It was therefore not possible to sample the cases as originally planned."<br><br>Page 21, lines 23-30.
**Reviewer 2 comments**			
**12**	Abstract:<br>Setting – more detail is needed here – Country? State? It says community care, but the title suggests it is people with dementia living at home? Please clarify<br>It is not clear if the EMBED-Care is a pre-existing framework – I suggest stating 'we previously developed…' if it was developed by the authors for feasibility evaluation. (I see this is mentioned in the strengths and limitations but it is not clear in the abstract)	Thank you for highlighting this point. The study recruited people living in their community in their own homes.<br><br>We have added information to the setting<br><br>We have also updated the description of the intervention.	"United Kingdom, National Health Services community care (people living with dementia at home)".<br><br>Page 2, lines 6-7.<br><br>"The intervention is the Empowering Better End of life dementia Care (EMBED-Care) Framework which we previously co-designed to enhance shared decision-making. The intervention comprises a holistic person-centred outcome measure known as the Integrated Palliative care Outcome scale for Dementia (IPOS-Dem), linked with clinical decision support tools (evidence-based approach to support decision making), priorities of care list (available in paper and digital formats), resources and training."<br><br>Page 2, lines 10-15.
**13**	Abstract:<br>19 – participants could be more specific (as per strengths and limitations)	Thank you for your request for more specific information regarding the participants. We have added updated this section.	"People living with dementia at home, their family carers and NHS community healthcare practitioners who provide care to people living with dementia".

			Page 2, lines 8-9.
**14**	Abstract: 31 – potential of the EMBED-Care framework to support SDM using the IPOS-Dem? Please be specific to clarify is this outcome part of the framework or is the outcome measure used to measure feasibility of the intervention?	We have added more information to describe the intervention and its components in more detail. The IPOS-Dem is a component of the intervention. The updated text now reads as:	"The intervention is the Empowering Better End of life dementia Care (EMBED-Care) Framework which we previously co-designed to enhance shared decision-making. The intervention comprises a holistic person-centred outcome measure known as the Integrated Palliative care Outcome scale for Dementia (IPOS-Dem), linked with clinical decision support tools (evidence-based, simple flow diagram of options to support decision making), priorities of care list (available in paper and digital formats), resources and training."<br><br>Page 2, lines 10-15.
**15**	Abstract:<br>33 - what are the clinical decision support tools in the framework? is not clear.	We have added more information in the intervention description to explain more about the IPOS-Dem and clinical decision support tools. Full detail is provided in the methods section.	"The intervention is the Empowering Better End of life dementia Care (EMBED-Care) Framework which we previously co-designed to enhance shared decision-making. The intervention comprises a holistic person-centred outcome measure known as the Integrated Palliative care Outcome scale for Dementia (IPOS-Dem), linked with clinical decision support tools (evidence based, simple flow diagram of options to support decision making), priorities of care list (available in paper and digital formats), resources and training."<br><br>Page 2, lines 10-15.
**16**	Abstract:<br>44 – Conclusion - The conclusion focuses on the outcome measure and support tools from the framework, but it is not clear what was used to determined feasibility of the intervention on shared decision-making and why it only mentioned some aspects of the framework? It is not clear what the framework includes and what the tools are. This	Thank you for highlighting this. We mentioned the aspects of the framework that were feasible. It would be inaccurate for us to describe the whole framework as feasible. We have updated the information in the methos of the abstract to detail what the clinical decision support tools are.	"The intervention comprises a holistic person-centred outcome measure known as the Integrated Palliative care Outcome scale for Dementia (IPOS-Dem), linked with clinical decision support tools (evidence based, simple flow diagram of options to support decision making), priorities of care list (available in paper and digital formats), resources and training."

	could be described in more detail in the intervention, if possible, with word count limits.		Page 2, lines 11-15.
**17**	Page 5<br>Background<br>8 – people suffering?	Thank you for raising this point. We have changed this to say 'health-related suffering' as it is the terminology used in the citation we referenced.	"Globally, it is projected that health related suffering will increase from 1.5 million in 2016 to 6 million by 2060".<br><br>Page 4, lines 2-3.
**18**	Page 5<br>Background<br>40 – can you give an example of a shared decision-making tool?	Thank you for requesting an example. By 'shared decision-making tool', we assume that the reviewer is requesting an example 'clinical decision support tool'. We have added this to this background.	"For example, a PCOM may highlight that a patient has pain, but a clinical decision support tool may help a clinician to consider potential causes of pain, particularly if the person cannot verbally communicate, but they have a family carer who can describe symptoms of the patient. Causes of pain may be dental, or pressure sore."<br><br>Page 4, lines 24.28.
**19**	Page 5<br>Background<br>45- as per my comment above, I suggest clear terminology between in-home resident of community care/living at home to avoid confusion.	We have kept the terminology of 'living at home' as it describes the setting of interest.	
**20**	Page 6<br>5 – this seems like a result that should not follow the aims in the background section. I suggest ending with the aim or describing the logic model above with an explanation that is also an aim to refine it.	Thank you for raising this point. Page 6 details the methods sections, not the background. We assume that the reviewer is meaning page 4.<br><br>One of the aims of the study was to refine the logic model from previous work where we developed the intervention that was evaluated in this study. We feel it is important to be transparent and make readers aware of this intention from the start. We have included this logic model refinement as an objective.	"The study objectives were: (1) To explore the processes of how (or not) the EMBED-Care Framework could enhance shared decision-making between people living with dementia (when able), family carers and healthcare practitioners. (2) To evaluate the feasibility (adherence and acceptability) of the EMBED-Care Framework to enhance shared decision-making (also referred to as 'the intervention' across this study). (3) Refine logic model from previous work that developed the intervention evaluated in this study."<br><br>Page 5, lines 6-12.

**21**	Methods<br>Design – these measures were not mentioned in the abstract and there could be greater focus on how feasibility of the intervention is evaluated.	Thank you for requesting more detail in the abstract. The measures used were primarily to be able to describe the characteristics of the participants recruited, as a component of demographic information. We did not use them as outcome measures to be compare changes over time. This is the reason the Edmonton Symptoms Assessment Scale and Clinical Dementia Rating scales were not mentioned. It was difficult to include much detail in the abstract due to the word limit of 300. We reported the most critical result in the abstract. We do describe this information in full in the methods section of the manuscript.	"Demographic data were collected, including age, gender, dementia severity (using Clinical Dementia Rating Scale - CDR),[38] to describe the participants within and across cases. The CDR is rated on a scale from 0 to 3. A score of 0 indicates no dementia, 0.5 indicates 'questionable' dementia, 1 indicates mild, 2 indicates moderate, and 3 indicates severe dementia. The CDR is a valid a reliable measure for assessing dementia severity.[38]<br><br>The Edmonton Symptom Assessment Scale (ESAS)[39,40] was used as an indicator of the level of needs an individual had, based on the number of symptoms experienced. The ESAS has nine symptoms; each rated on a scale from 1 (no symptom) to 10 (worst symptom possible). For each symptom, a score of ≤3 is considered stable, 4-6 requires further assessment, and ≥7 is unstable. We converted the number of symptoms that fall within each of these three categories into a score out of 100, using the ESAS guidance. This figure is reported in Table 1."<br><br>Page 11, lines 16-26.
**22**	Page 7<br>- The text size seems inconsistent<br><br>5 – this could be more concise. It could be more specific if it is talking about the co-design process of developing the framework or the intervention itself. Sub heading may help to describe each of the components of the intervention and what it involves.	Thank you for raising this, we have fixed the inconsistencies.<br><br>Thank you for your suggestion to improve the clarity and flow of the intervention described. Page 7 is of figure 1. We assume the reviewer is referring to page 6. This section largely describes the intervention that was evaluated in this study. We have split up the intervention components and describe them a more detail.	1. "A holistic person-centred outcome measure (the IPOS-Dem) to identify symptoms and concerns[21,22] (referred to as needs in this study). The IPOS-Dem is a PCOM, initially designed as a staff proxy measure for people with dementia living in care homes. It comprises multiple health domains, including physical, psychological, and social

	7 – see comment above for clarifying setting.	We have updated the details of the eligibility criteria for clarity.	wellbeing, and asks about family concerns.[21,22]<br><br>2. The IPOS-Dem items were each linked with evidence-informed clinical decision support tools to inform management of care and treatment and aid decision-making.[28] Clinical decision support tools, known as 'rules of thumb' or 'heuristics', is a method for presenting the best available options to aid decision-making on care and treatment. They are displayed in simple flow diagrams to help consider possible options.<br><br>3. The priorities of care list was designed to identify and inform understanding on what mattered most to the person and/or family to prioritise and plan care and treatment."<br><br>Page 6, lines 9-25.<br><br>"People living with dementia in their own home."<br><br>Page 9, lines 25.
**23**	Figure 1 – I like the visual representation, but this is difficult to follow. Maybe some numbers or subheadings could help direct more intentional order to what should be viewed first/flow of the visual that can be followed or make it clear what the arrows mean? It may need to be simplified into the elements of the intervention separated from the elements of shared decision-making?	Thank you for requesting clarification regarding figure 1. Due to this figure being already published, we are unable to modify it. Within the figure, there is a 'key' to describe the components of the figure. Additionally, a table with the full intervention component, informed by the TiDIER guidance is detailed in the appendices. We have updated the text in the manuscript to provide more clarity on what figure one shows.	"The shared decision-making intervention is illustrated in figure 1. This figure is from our co-design study[1] and shows how the intervention works to enable shared decision-making. The shared decision-making elements is informed by the Enriched Model of Collaborative Deliberation.[8] A table with full intervention detail, informed by the Template for Intervention Description and Replication Checklist (TIDieR)[30] is reported in the appendices."<br><br>Page 7, lines 3-7.

**24**	Page 9<br>- Please check text size throughout<br><br>- Try to avoid crossing between the previous study and this study too much. Unless I missed it, this is the first mention of interviews? Are they part of this study? It would be helpful to include this in the abstract or definition of how feasibility was planned to be evaluated in the abstract methods. Or results of how many were conducted and with whom<br>Setting – in the United Kingdom? A community team helping people with dementia living in their home?	Thank you for raising this, we have fixed the inconsistencies.<br><br>This section focuses on the study design and data collection for this study. The interviews were an aspect of this study. Please note that the guidance for the abstract structure does not allow us to detail the data collection and analysis. However, we do detail this in the methods sections of the manuscript.<br><br>We have updated the abstract to include the 'United Kingdom' and 'home care setting'.	Page 10, lines 23-28.<br><br>"**Design**: A mixed methods feasibility study and process evaluation using a multiple case study design at the individual level of person with dementia, family carer and practitioner.<br><br>**Setting**: United Kingdom, National Health Service community care (people living with dementia at home).<br><br>**Participants**: People living with dementia at home, their family carers and NHS community healthcare practitioners who provide care to people living with dementia.<br><br>**Intervention**: The intervention Empowering Better End of life Dementia Care (EMBED-Care) Framework which we previously co-designed to enhance shared decision-making. The intervention comprises a holistic person-centred outcome measure known as the Integrated Palliative care Outcome Scale for Dementia (IPOS-Dem), linked with clinical decision support tools (evidence based approach to support decision making), priorities of care list (available in paper and digital formats), resources and training.

			**Results**: The study was made up of three cases, comprising a person living with dementia at home and/or their family carer and a practitioner."<br><br>Page 2, lines 6-17.<br><br>"We incorporated a process evaluation to understand *how* and *why* the intervention worked (or not) for people affected by dementia (including people with dementia and their family carers) at home…"<br>Page 5, line 22-23.
**25**	Results<br>Page 11<br><br>- Please be more specific – 12 people? Is this everyone or people with dementia etc	Thank you for seeking clarity regarding the number of people assessed for eligibility. We have updated this section for clarity. These numbers are also detailed in figure 1.	"The study was conducted from February to April 2024 in the participating community NHS Trust. Twelve people (including people living with dementia (n-4), their family carers (n=4) were assessed for eligibility, and eight were recruited to form three cases (Figure 1)."<br><br>Page 12, lines 12-14.
**26**	Page 14<br><br>- I suggest describing a summary of table 11 then just have '(Table 11)' should this be table 1?<br><br><br><br><br>- There needs to be a description of the scales used and scoring for each and definitions of Alzheimer's etc	Thank you for highlighting this typo. We have updated the table to the correct number. It is table 1.<br><br><br><br><br><br>There is a summary under participant characteristics as this is what the table alludes to.	"Table 1: Participant characteristics"<br><br>Page 14, line 9.<br><br><br><br><br>"The CDR is rated on a scale from 0 to 3. A score of 0 indicates no dementia, 0.5 indicates 'questionable' dementia, 1 indicates mild, 2 indicates moderate, and 3 indicates severe dementia. The CDR is a valid a reliable measure for assessing dementia severity.[38]"<br><br>"The ESAS has nine symptoms; each rated on a scale from 1 (no symptom) to 10 (worst symptom possible). For each symptom, a score of ≤3 is considered stable, 4-6 requires further

			assessment, and ≥7 is unstable. We converted the number of symptoms that fall within each of these three categories into a score out of 100, using the ESAS guidance. This figure is reported in Table 1."<br><br>Page 11, lines 15-26.
**27**	Page 17<br><br>- A summary of text should be provided describing the themes and subthemes before they are presented. As mentioned above it did not seem clear that interviews and themes were part of this study.<br><br>- This section is too long and should be more concise. I suggest using an appendix.	We have added a summary of the themes.<br><br>Thank you for your suggestion to use appendix to reduce the content of the results section. However, the detail provided in the manuscript are key to the flow and understanding of our research. By reducing this section, we think believe the clarity of the reporting would be compromised. We believe also believe that it would be easier to keep the content in the main manuscript so that readers do not need to go elsewhere for key information.	"Two themes were generated from the qualitative data for how the intervention worked to enhance shared decision-making. 1) Theme 1: Establishing a relationship to build knowledge of the person and engage with the person's network (including the family carer and healthcare practitioner). 2) Theme 2: Healthcare practitioner training and support to use the shared decision-making intervention. Page 12, lines 17-21.
**28**	Discussion<br>Page 20<br><br>8 – The findings from this study indicate that intervention could enhance some elements of the intervention.' – this does not make sense? Two elements?<br><br>18 – perhaps a summary of what change in the logic model would be more helpful?	Thank you for raising this. We have corrected this.<br><br>We have added a summary of what changed in the logic model. We have also updated the logic model itself to correct for typo.	"The findings from this study indicate that some elements of the intervention could enhance shared decision-making. These were the IPOS-Dem and clinical decision support tools. The priorities of care list require further revision to increase the likelihood of use."<br><br>Pages 20, lines 3-6.

			The main findings are depicted in orange text in Figure 2. For example, the IPOS-Dem and Clinical Decision Support Tool were key components of the intervention that were used, however the priorities of care list was not. These intervention components, designed for use digitally and in paper form, linked with mechanisms of impact and shared decision-making, such as 'knowing the person' through using the IPOS-Dem. Whereas, 'engaging and considering the perspectives of the person and family carer to determine priorities for care...' was managed using the Clinical Decision Support Tools.<br><br>Page 20, lines 12-18.
**29**	Conclusion<br>- As above, for someone not familiar with the framework, each element should be described for better understanding with a clear focus on the evaluation of the feasibility across three case studies of the framework. For example, it would be helpful to describe how the outcome measure in the framework is intended to facilitate shared decision-making. E.g. who delivers the outcome measure in the framework, how was it delivered? What does it consist of? And how does it facilitate shared decision making? And then a conclusion of how this was shown to be feasible e.g. interviews with who? suggested what? This could be expanding on the sentence "The IPOS-Dem facilitated conversations between people living with dementia and/or their family carers and nursing assistants" but while an outcome may identify needs how does it lead to shared-decision making? And how did it facilitate conversations? Between who? And were	Thank you for requesting clarity how the IPOS-Dem and clinical decision support tool facilitated shared decision-making. We have added additional information to describe this.	"The IPOS-Dem facilitated conversations between people living with dementia and/or their family carers and nursing assistants through shared completion of the IPOS-Dem between the person and/or their family carer, and practitioner, to identify symptoms and concerns of the person and family carer over the past week. Once the IPOS-Dem was completed, clinical decision support tools, linked with symptoms on the IPOS enabled discussion between people practitioners and people living with dementia and/or their family carers regarding exploration of preferences for care and treatment. This decision was a collaborative effort. The results indicate that the intervention, particularly the IPOS-Dem could be used by nursing assistants who could report to registered nurses, thus fostering a thorough care process."<br><br>Pages 24, lines 4-12.

	conversations management decisions? Or did this mean patient values and preferences were identified? Was this from interview findings? Observations?		

VERSION 2 - REVIEW

**Reviewer**	**1**
**Name**	**Jones, Steven**
**Affiliation**	**University of Chester, Public health school**
**Date**	**01-Sep-2026**
**COI**	

Improved read and clarity on second draft. Good use of appendices and supporting documents. Strengths and limitations addressed, and how sampling issues overcome that could have impacted significantly (pragmatically). Focus on what the investigation team might change or do different next time, to mitigate method shortcoming .The paper should provoke thought and debate. I wonder how barriers to clinical uptake/ implementation may progress in future study designs.

VERSION 2 - AUTHOR RESPONSE

Reviewer comment			
**6**	Improved read and clarity on second draft. Good use of appendices and supporting documents. Strengths and limitations addressed, and how sampling issues overcome that could have impacted significantly (pragmatically). Focus on what the investigation team might change or do different next time, to mitigate method shortcoming. The paper should provoke thought and debate. I wonder how barriers to clinical uptake/ implementation may progress in future study designs.	We thank the reviewer for their on the original manuscript and for taking the time to review the revised version.<br><br>A major consideration going forward is ethnic diversity and a wider range of dementia severity which may or may not impact on uptake. This is a priority area for future study. We detail this plan in the discussion section of the manuscript.	Page 21, lines 32-33 and page 22 lines 1-4.
