# Supplementary material for: A mixed methods feasibility evaluation of a shared decision-making intervention for community dwelling adults living with dementia, family carers and practitioners
Source: BMJ Open. 2026 Oct 28;16(10):e121790. doi: 10.1136/bmjopen-2026-121790 (PMC13723839; doi:10.1136/bmjopen-2026-121790)
Supplement: Supplementary file 2 [file bmjopen-16-10-s001.docx]

Appendix 1: COREQ checklist

| No | Item | Guide questions/description | Page/line numbers |
| --- | --- | --- | --- |
| **Domain 1: Research team and reflexivity** | | | |
| 1 | Interviewer/facilitator | Which author/s conducted the interview or focus group? | Page 10, lines 26-27 |
| 2 | Credentials | What were the researcher's credentials? E.g. PhD, MD | Page 10, lines 26-27 |
| 3 | Occupation | What was their occupation at the time of the study? | Page 10, lines 26-27 |
| 4 | Gender | Was the researcher male or female? | Page 10, lines 26-27 |
| 5 | Experience and training | What experience or training did the researcher have? | Page 10, lines 26-27 |
| Relationship with participants | | | |
| 6 | Relationship established | Was a relationship established prior to study commencement? | Page 9, lines 15-16 |
| 7 | Participant knowledge of the interviewer | What did the participants know about the researcher? e.g. personal goals, reasons for doing the research | No other knowledge of the researcher except for in the capacity of the study consent process and for data collection.  Page 10, line 13 |
| 8 | Interviewer characteristics | What characteristics were reported about the interviewer/facilitator? e.g. Bias, assumptions, reasons and interests in the research topic | The researcher had experience of data collection with people living with dementia and their family carers.  Page 10, lines 26-27 |
| **Domain 2: Study design** | | | |
| Theoretical framework | | | |
| 9 | Methodological orientation and Theory | What methodological orientation was stated to underpin the study? e.g. grounded theory, discourse analysis, ethnography, phenomenology, content analysis | Page 5, lines 15-32 and page 6, lines 1-9. |
| Participant selection | | | |
| 10 | Sampling | How were participants selected? e.g. page 9, lined , convenience, consecutive, snowball | Page 9, line 18-22. |
| 11 | Method of approach | How were participants approached? e.g. face-to-face, telephone, mail, email | Page 9, lines 23-32 and page 10, lines 1-21. |
| 12 | Sample size | How many participants were in the study? | Page 12, lines 12-17.  Page 13 – figure 2 (PRISMA flow diagram). |
| 13 | Non-participation | How many people refused to participate or dropped out? Reasons? | Page 13 – figure 2 (PRISMA flow diagram). |
| Setting | | | |
| 14 | Setting of data collection | Where was the data collected? e.g. home, clinic, workplace | Page 9, line 26.  Page 10, lines 8-9. |
| 15 | Presence of non-participants | Was anyone else present besides the participants and researchers? | N/A |
| 16 | Description of sample | What are the important characteristics of the sample? e.g. demographic data, date | Page 12, lines 10-20. |
| Data collection | | | |
| 17 | Interview guide | Were questions, prompts, guides provided by the authors? Was it pilot tested? | Page 9, lines 7-8. |
| 18 | Repeat interviews | Were repeat interviews carried out? If yes, how many? | N/A |
| 19 | Audio/visual recording | Did the research use audio or visual recording to collect the data? | Page 11, lines 1. |
| 20 | Field notes | Were field notes made during and/or after the interview or focus group? | N/A |
| 21 | Duration | What was the duration of the interviews or focus group? | Page 11, lines 1. |
| 22 | Data saturation | Was data saturation discussed? | N/A |
| 23 | Transcripts returned | Were transcripts returned to participants for comment and/or correction? | N/A |
| **Domain 3: analysis and findings** | | | |
| Data analysis | | | |
| 24 | Number of data coders | How many data coders coded the data? | Page 11, lines 15-18. |
| 25 | Description of the coding tree | Did authors provide a description of the coding tree? | Supplementary file, appendix 3. |
| 26 | Derivation of themes | Were themes identified in advance or derived from the data? | Page 11, lines 1-14. |
| 27 | Software | What software, if applicable, was used to manage the data? | Page 11, line 4. |
| 28 | Participant checking | Did participants provide feedback on the findings? | No |
| Reporting | | | |
| 29 | Quotations presented | Were participant quotations presented to illustrate the themes / findings? Was each quotation identified? e.g. participant number | Page 16, lines 9-11.  Page 16, lines 20-23.  Page 16, lines 29-31.  Page 17, lines 9-11.  Page 17, lines 15-17.  Page 17, lines 25-26.  Page 17, lines 30-31.  Page 18, lines 1-2.  Page 18, lines 7-10.  Page 18, lines 14-16.  Page 18, lines 21-23.  Page 19, lines 5-8.  Page 19, lines 14-16.  Page 19, lines 21-25. |
| 30 | Data and findings consistent | Was there consistency between the data presented and the findings? | Yes |
| 31 | Clarity of major themes | Were major themes clearly presented in the findings? | Page 15, lines 3-4.  Page 17, lines 18-19. |
| 32 | Clarity of minor themes | Is there a description of diverse cases or discussion of minor themes? | N/A |

Appendix 2: Shared decision-making intervention, incorporating the EMBED-Care Framework

| **EMBED-Care Framework component name** | **Why? (rationale/theory/goal)** | **What? (Materials and procedures)** | **Who provides? (Background, training etc)** | **How?** | **Where? (The location where the intervention occurred)** | **When and how much** | **Tailoring** |
| --- | --- | --- | --- | --- | --- | --- | --- |
| **Integrated Palliative Outcome Scale for Dementia (IPOS-Dem)** | **Shared decision-making**  Identification of symptoms and concerns. | The person with dementia or their family carer (or both together) completes the IPOS-Dem before a practitioner visit. More than one version of the IPOS-Dem may be completed. The IPOS-Dem is completed monthly or more frequently if required. This should be agreed on this at the initial appointment. | The researcher trained the clinical team, including champions who were nursing assistants. The champions supported the rest of the clinical team to use the intervention and train new staff members. Champions could be nursing assistants, band 6 palliative care or Admiral Nurses, or a Band 7 senior palliative care nurse. | Face to face/telephone.  App/web-based. | In the home of the person living with dementia/their family carer or remotely over the phone. | The minimum use is monthly but can be completed more frequently as clinically indicated. | How the IPOS-Dem is completed, and its frequency can be agreed upon between the person, family carer and health and social care professional at the first meeting and reviewed as required.  Paper version of the intervention or app/web-based platform can be used depending on preference.  If using the app/web-based version of the intervention, the app will automatically generate alerts for items on the IPOS-Dem that score 1 and above. Alerts can be muted or adapted based on clinical need and judgment. |
| **Clinical decision support tools** | **Shared decision-making**  The clinical decision support tools were resources to support decision-making. The clinical decision support tools were linked to the items on the IPOS-Dem (e.g. pain, eating and drinking, etc). The clinical decision support tools were automatically generated for any IPOS-Dem item that score ≥1. They were intended to be reviewed with the person and/or their family carer so that they understand the options available to them and be able to discuss with a practitioner about the options and their preferences for care and treatment. | Twelve decision support tools are available for people with dementia, practitioners and family carers. The decision support tools support decision-making around care and treatment.  The decision support tools were:   1. Spiritual, identity and well-being; 2. Delirium; 3. Eating, drinking and mouthcare; 4. Pain; 5. Skin care; 6. Sleep; 7. Constipation and incontinence; 8. Weakness and lack of energy; 9. Mobility; 10. Maintaining emotional and psychological well-being; and 11. Carer well-being.   The alerts prompted relevant clinical decision support tools to support discussion and care and treatment decisions between the person with dementia/ carer/ and health and social care practitioner. Decision support tools may suggest escalation if previous care plans have not been effective. | The researcher trained the clinical teams to use the intervention. These were nursing assistants or admiral, or palliative care nurses can use this intervention component.  The nursing assistants complete the assessment were required to review the decision support tools and escalated any concerns to the nurses they are paired with. | This can be used face to face or over the telephone with a patient. | In the home of the person living with dementia. | Each time the IPOS-Dem is completed, the clinical decision support tools are expected to be used, but they can be used at any time. They may be used less frequently as participants become familiar with it. | Paper copy of the intervention or app/web-based platform can be used depending on preference.    People with dementia, family cares and practitioners can access the decision support tools at any time, by themselves or together as a group.  The relevant clinical decision support tools are flagged up by the app automatically depending on the symptoms affecting the person with dementia. |
| **Priorities of care list** | **Shared decision-making**  The person with dementia and their family carer discusses their priorities for care and the treatment/care options to address the symptoms and concerns raised by the IPOS-Dem. | The priorities of care list follows on from the IPOS-Dem and review of the clinical decision support tools.  A priority list is created or summarised, guided by the IPOS-Dem and reviewing the decision support tools.  The priorities of care list section of the app is a free text box where people with dementia and/or family carers can detail main symptoms they wish to address and plans to do so. Users could freely enter symptoms and plans to address the symptoms. | The researcher trained members of the community clinical teams to use the intervention. These were nursing assistants, Admiral nurses or palliative care nurses | The priorities of care list can be used face to face or over the telephone with a patient using paper or app version of the intervention. | In the home of the person living with dementia/their family carer. | Each time the IPOS-Dem is completed. | The method to determine priorities of care can be adapted to the existing clinical procedure. |
| **Training and support** | **Shared decision-making using the EMBED-Care Framework**  Comprehensive training and refresher training was provided to all staff | Initial face-to-face and virtual training on the using the Shared decision-making intervention and demonstration of the digital version of the intervention.  Refresher training provided when required  Digital support (videos on the app, including IPOS-Dem, decision support tools, shared decision-making, and escalating concerns)  Email/Telephone helpline | The researcher provided comprehensive training to all staff initially, then refresher training to champions and again to all clinical staff. The research team set up biweekly meetings with the champions  The role of champions was to address any queries that colleagues had, encouraging and motivating colleagues, and addressing any team or feeding back queries to the research team for resolution. | A combination of face-to-face and virtual training. | Face-to-face or virtual training at the Community NHS site. | Pre and post implementation of intervention. | A Refresher trainer was provided when needed. |

The Template for Intervention Description and Replication (TIDIER) checklist^30^ was used to structure the reporting of the study intervention

Note: PwD = person with dementia, IPOS-Dem = Integrated Palliative Outcome Scale for Dementia, EMBED-Care = Empowering Better End of life Dementia Care, PCOMs = person centred outcome measures

Appendix 3: coding tree

The EMBED-Care Framework for Shared decision-making developed from previous study to inform the evaluation of this study

| **Main areas** | **What?** | **How?** |
| --- | --- | --- |
| Knowing the person and personalisation of care | Personalisation of care | Involvement of people with dementia and family carer to enhance understanding of their needs across health domains  During or after a visit with the person with dementia and family  IPOS-Dem supports the identification of needs to inform treatment and care  Use of multiple versions of the IPOS-Dem  Aids discussion and enables confidence to communicate what is important to the person and family, and contribution to care and treatment decisions  Consideration of individual culture/beliefs as impacting willingness to engage |
|  | Flexibility in the use of the EMBED-Care Framework is key to the personalisation of care and impacting shared decision-making | The frequency of IPOS-Dem use and format (digital or paper) are tailored to the person with dementia. How the IPOS-Dem is used is to be tailored to individuals.  Practitioners required flexibility too, in terms of using the intervention either as part of a conversation with the person or asking people with dementia/family carers to complete the IPOS-Dem |
| Engage with and consider the perspectives of all involved in shared decision-making |  |  |
|  | Sensitivity to the needs raised by the person with dementia and family carer | Requires engagement from the person and family carer |
|  | Accessible language | Enables engagement from the person and family carer in the decision-making process |
|  | Managing multiple priorities, care and treatment preferences | Practitioner role is to steer conversation and allow opportunities for person and family carer to be heard |
| Evaluation of decision-making (creating or adapting existing care plans) |  |  |
|  | Monitoring and review | Reviewing as frequently as needed with intention of adapting/modifying existing care plans if required |
| Implementation requirements |  |  |
|  | Paper intervention to complement digital intervention | Technology to aid use of intervention but could as a barrier to the patient-practitioner interaction. Paper intervention will help with this. |
|  | Ease of use | Quick to complete and simple to understand |
|  | Busyness, heavy workload and time constraints | Avoid duplication of work which adds to workload |
|  | Organisational leadership and support | Champions at different levels of the organization |
|  | Support for people with dementia and family carers to understand the intervention | Information provision in ‘bite-size chunk’  Animated videos to aid understanding of intervention  Training may help some – email and telephone helpline |
|  | Training | On all the components of the intervention, including communication  Use of comprehensive and quick manual/guides to support training and intervention implementation |
|  | Familiarisation with the intervention | Time to familiarise self with the intervention required to understand the intervention and support people with dementia and family carers |
| Anticipated benefits |  |  |
|  | For staff – impact on patients – more than just a job, skills development.  For family carers – empowerment to communicate needs and priorities and engagement with practitioners in decision-making |  |

Appendix 4: case summaries

Case One Summary

**Demographics**

**Person with dementia**

Clinical Dementia Rating Scale: 0.5 (mild dementia)

Edmonton Symptom Assessment Scale total score: 34 (out of 100), although four symptoms received a score of five or more. These were tiredness, drowsiness, shortness of breath and wellbeing.

**Case 1 Edmonton Symptom Assessment score for each item**

| Pain | Tiredness | Drowsiness | Nausea | Lack of appetite | Shortness of Breath | Depression | Anxiety | Wellbeing |
| --- | --- | --- | --- | --- | --- | --- | --- | --- |
| 1 | 8 | 7 | 1 | 4 | 6 | 1 | 1 | 5 |

**Family Carer**

Relationship between the person with dementia and family carer: husband/wife (family carer)

**Practitioner**

Role: Nursing Assistant

Number of years of clinical experience: 10+ years

**Summary of how the case used the intervention**: A combination of digital and paper intervention was used. The person and the family carer (referred to as dyad) were given a paper copy of the IPOS-Dem at the first visit, as this was their preference. The clinical decision support tools were printed and given to the dyad a few weeks later. The practitioner used the digital version of the intervention, directly inputting responses from the IPOS-Dem into the app. The dyad looked at the paper copy of the IPOS-Dem during the home visit and followed along with the practitioner. The IPOS-Dem was completed together by the person with dementia, family carer and practitioner (nursing assistant) The decision-support tool and priorities of care list were unused. The intervention was used twice during a two-month period at timepoints one and two.

**Objective 1: To explore the processes of how the EMBED-Care Framework enhances shared decision-making for people affected by dementia living at home and practitioners**

Establishing rapport is an important step in using the EMBED-Care Framework. The relationship between the practitioner, family care and the person with dementia was central to the dynamic of the triad and interaction during a home visit. Once rapport was established, the IPOS-Dem was used as a conversation starter and a source for further discussions.

Direct quotes

…but then it, it's quite good, because I know both families that I'm using it with, so we've got that rapport already, so I think that helped. – **practitioner**

Because we've got to know each other a bit more now, you see, and [the practitioner] obviously is getting an idea of what sort of people we are, and vice versa, you see, and it is, it gets more relaxed. – F**amily carer**

I agree. – **Person with dementia**

The IPOS-Dem was used to identify the needs (referred to as symptoms and concerns) of the person with dementia. The participants in this case felt it was important to involve the person with dementia and the family carer from the beginning. The dyad relied on each other to recall the person's needs. The person with dementia was in the milder dementia stage and felt that they coped well with being involved in the discussion about their needs. The carer valued the opportunity to be involved in the discussions around the person’s needs and the dyad used the paper versions of the IPOS-Dem questionnaire during this stage, whilst the practitioner used the digital version of the intervention.

Direct quotes

I thought it was very interesting and we did discuss some of the questions and some were asked. – **person with dementia**

…and the couple that were the actual person living with dementia and [the family carer], and they used a paper version. So it was quite good, it's been quite nice, actually, doing it and getting going. - **practitioner**

Well, we, what we did, well, I filled in, as we were doing it I filled in a paper copy of what [the practitioner] was putting down. I think [the practitioner] was on a computer, wasn't she? **– family carer**

Erm... I think we just had a look at it on the laptop all together, and like took a lot of lead from the family that were there, and kind of said, oh, what's that about, what's that about? So we kind of just went into it then and when it was more family-led or carer-led, me leading it at that point. – **practitioner**

The dyad used the IPOS-Dem to reflect on the extent to which symptoms were affecting the person with dementia, therefore enabling the person, the family carer and practitioner to reflect and discuss and valuing the opportunity to talk through the different symptoms and concerns that the person with dementia was experiencing. The dyad discussed with the practitioner to identify symptoms, priorities, treatment and care options for the person with dementia. The dyad was proactive in identifying care and treatment preferences that they wished to implement, informed by the clinical decision support tools. The practitioner felt that a review of the decisions made was necessary for monitoring the needs of the person with dementia.

Direct quotes

I think where we've used it [referring to the IPOS-Dem] before, and we have been speaking to like, obviously, the families and how it affects them, but going through it separately with them, they were almost grading the person worse, the symptoms worse than we would have graded them, as such, but that's really good, that was really insightful for me to see, obviously, that it's having more of an impact on that person than you would have kind of necessarily assigned to it. – **practitioner**

I can't think of any particular ones. Erm... no, here's one here I'm looking at now. Diarrhoea, now we wouldn't have thought of anything like that coming into the equation, you see, so that's a typical example of the odd thing that we wouldn't associate with what we were dealing with. – **family carer**

because we've talked, we will talk about the subject of the question and then between us we would, we would say straightaway, without hesitation, oh, slightly or not at all, but when it got to moderately or severely – **family carer**

It was funny, actually, because I said about, I said this to them, I said, what is going to change between either of your opinions, and they were like, no, we'll just discuss it through and work out whether, so they were quite good about saying, actually, so the person living with dementia said, oh, I reckon that's only a like slightly to moderately, and the carer was like, no, it's severely *[laughs]*, moderately to severely rather than slightly to severely, because that's affecting you more than you... more than you remember. - **practitioner**

For the practitioner, training sessions that incorporated a combination of teaching and practical demonstration aided knowledge acquisition about the intervention and its use. Although time for familiarisation with the intervention was required before use with the dyad. Additionally, resources such as short guides in digital and paper formats were reported to be helpful.

Direct quotes

I think it has been helpful, it seems quite spaced out from when we first did it way back when, when you were introducing it to us and then again a bit later at [mentioned hospital location] – **practitioner**

No, I think, like I say, the most helpful thing was having that practise account to be able to practise on, so that might be something to incorporate into a training session, so, when you do a training session, get everybody to sign into the practise account and then run through how to do it while they're actually doing it. – **practitioner**

No, I think it, to be fair, the training was good, and we've kind of learnt, and then the follow-up afterwards that we've had has been really good. – **practitioner**

So I'm hoping that the more we do it with people, the more they'll get used to the system and they'll be like, oh, yeah, that's that, that's that, that's that. And we can kind of fly through it a little bit, so, yeah. – **practitioner**

The practitioner valued simplicity, and the protected time from team leadership was necessary to not rush the use of the intervention during home visits. An important point highlighted by the person with dementia. Adequate time was necessary for the person with dementia to feel comfortable during and to engage in its use with the practitioner during the home visit. However, the practitioner felt that some of the intervention components, particularly the clinical decision support tool, was overwhelming due to the amount of information generated and the time required to go through it.

Direct quotes

Yeah, I don't know if you could have like an even more simplified one, so it's literally just got the [IPOS-Dem], rather than having lots of different buttons on it, that are great for us to use, but it would be good if you kind of had a simplified version for people that aren't very techy. – **practitioner**

Meaning myself, you know, don't, don't try and get it in too quickly. Gently go into it each time, you see, otherwise you can, it starts getting scrambled up like an egg. – **person with dementia**

For me, no, I'm fine, yeah, I have no problem in things like that, I can discuss things forever. It's just that, like [the person with dementia] says, if it's, if it goes on for too long then I think I'm just reiterating myself now – **family carer**

I'll quite happily flick through everything to learn what it's about and I think some people see that as time consuming, whereas if they'd got a simple version with just the bit that they've got to do, without everything else, they could just go to it, do it and be done. So I think having the option for either like the more technical version or the simplified version for staff would be a really good option. – **practitioner**

All participants in the case raised the need for flexibility in how the intervention is configured for use, such as having the option to use it together during a visit, or the person and the family carer to use it prior to a visit by the practitioner.

Direct quotes

but if it had been sent to them ahead of time, they could have at least had the opportunity to familiarise themselves with it, and make little notes on it – **practitioner**

Yeah, [the practitioner] should come round… Because we can, any points, we can raise them straightaway and be clear, clear about what they're really getting at and want to know. – **person with dementia**

You know, we've got extra copies to do this with, so I would be okay assessing, if you like, what's going on and what's relevant at the time and filling it in, I would be happy with that. **– family carer**

because we've talked, we will talk about the subject of the question and then between us we would, we would say straightaway, without hesitation, oh, slightly or not at all, but when it got to moderately or severely**– family carer**

The family carer felt the EMBED-Care Framework to aid communication of needs, whilst the practitioner felt that the EMBED-Care Framework enhanced communication between the practitioner and other clinical colleagues. Additionally, the involvement of the dyad encouraged them to take ownership of the person’s care and be proactive in implementing different care and treatment solutions, as well as symptom monitoring.

Direct quotes

Yeah, he agrees, like he just said, he agrees with that, because you weigh people up, don't you, and you know what type of person you're dealing with, because everybody's different. So, and I think both sides of the conversation are very relaxed, I don't think either party, or either [the practitioner] or us as one item, feel as though we're restrained in any way, you know, as to what we say or discuss. – **family carer**

I think it's really good for them, because when we were looking through it on the laptop, some of the bits [the clinical decision support tools], they were going, oh, that's a really good idea, that's a good, we'll try that. So it was giving them ideas on how they can be proactive in relieving symptoms at home without needing support from a medical professional, some of the bits on there they could do themselves already and get going with, so I think it's definitely good in that respect. – **Practitioner**

**Objective 2: The feasibility (adherence) and acceptability of the EMBED-Care Framework to enhance shared decision-making.**

In this case, the intervention was used once in T1 and once in T2. All the IPOS-Dem items were completed on all three occasions with no missing items (see below). There were no incomplete items on the IPOS-Dem when used. The ‘cannot assess” option was not used.

**Case one IPOS-Dem radar plots**

|  | T1 | T2 |
| --- | --- | --- |
| Sleeping problems | 0 | 0 |
| Diarrhoea | 0 | 0 |
| Hallucinations | 3 | 1 |
| Agitation | 1 | 0 |
| Wandering | 0 | 0 |
| Eye pain* | 1 | 0 |
| Feeling anxious or worried | 1 | 2 |
| Family’s anxiety and worry | 2 | 3 |
| Feeling depressed | 0 | 0 |
| Loss of interest in usual activities | 0 | 3 |
| Feeling at peace | 1 | 2 |
| Positive interactions with others | 0 | 2 |
| Have practical problems been addressed? | 0 | 1 |

|  | T1 | T2 |
| --- | --- | --- |
| Pain | 2 | 3 |
| Shortness of breath | 1 | 2 |
| Weakness or lack of energy | 3 | 3 |
| Nausea | 0 | 0 |
| Vomiting | 0 | 0 |
| Poor appetite | 0 | 0 |
| Constipation | 0 | 0 |
| Dental problems | 0 | 2 |
| Sore or dry mouth | 2 | 3 |
| Drowsiness (sleepiness) | 3 | 3 |
| Poor mobility | 2 | 2 |
| Swallowing problems | 1 | 0 |
| Skin breakdown | 0 | 0 |
| Difficulty communicating | 1 | 1 |

*Note: lower score is interpreted as less severe symptoms

Utility questionnaire (detailed below) responses of how the intervention was used

The family carer, although they had not seen or used the clinical decision support tool, had a positive attitude towards the idea and felt it was a tool that could be helpful. Although the practitioner highlighted the need for more time to become familiar with the whole intervention and to navigate the contents of clinical decision support tools.

Direct quotes

Yeah, that would be good, yeah, yeah, a support tool like that would be helpful, yeah, because that gives more explanation, more in detail, doesn't it. – **family carer**

I think the biggest thing is it, because of, if you've got more than one symptom it kind of throws a lot of information back and it's picking up, it's almost skimming out what you don't need a little bit, but I think that'll just come with time, as we use it more we'll just get used to picking out the relevant bits that people can do something about – **practitioner**

Overall, the intervention was well received by the person with dementia and the family carer who found it “helpful” in providing detailed information on symptoms highlighted by the IPOS-Dem as causing concern. For the practitioner, features such as ‘reminders’ were helpful in ensuring monitoring of the person with dementia and continuity of care by handing over to colleagues when appropriate.

Direct quotes

No, everything seemed to be straightforward, because, as I say, as we were discussing each question we made sure that I fully understood it. – **person with dementia**

I do think it's really good because, obviously, if I needed to, if for some reason I didn't visit within that set time of needing to do it, it would remind me that it needed doing, and I could always hand that over to one of the other managers for that person… - **practitioner**

Case two summary

**Demographics**

**Person with dementia**

Clinical Dementia Rating Scale: 2 (moderate dementia)

Edmonton Symptom Assessment Scale score: 45 (out of 100), although four symptoms received a score of five or more. These were tiredness, drowsiness, lack of appetite and wellbeing.

**Case 2 Edmonton Symptom Assessment score for each item**

| **Pain** | **Tiredness** | **Drowsiness** | **Nausea** | **Lack of appetite** | **Shortness of Breath** | **Depression** | **Anxiety** | **Wellbeing** |
| --- | --- | --- | --- | --- | --- | --- | --- | --- |
| **4** | **8** | **8** | **2** | **7** | **2** | **3** | **4** | **8** |

**Family Carer**

Relationship between the person with dementia and family carer: husband/wife (Peron with dementia)

**Practitioner**

Role: Nursing Assistant

Number of years of clinical experience: 10+ years

**Summary of how the participants, in this case, used the intervention**: the EMBED-Care Framework app was used between the practitioner and the family carer. The person with dementia, although present, was not able to contribute to care and treatment decision-making. The EMBED-Care Framework was used three times over a two-month period: two weeks apart in T1 (4 weeks after baseline) then again at T2 (8 weeks from baseline). The IPOS-Dem was completed together by the family carer and practitioner (nursing assistant) during a home visit, and the clinical decision support tools were reviewed together. The priorities of care list was used once in the second month after recruitment. The practitioner appreciated this is new and anticipated subsequent visits to be much quicker.

**Objective one – to explore the processes of how the EMBED-Care Framework enhances shared decision-making for people affected by dementia living at home and practitioners**

Data from the qualitative highlighted rapport building as the first step in using the EMBED-Care Framework together. The practitioner felt it was important to know the person with dementia and the family carer well, and for the family carer to be involved in using the EMBED-Care Framework from the first home visit. The practitioner completed the IPOS-Dem with the family carer to identify the needs of the person with dementia and ensure that nothing was missed. The EMBED-Care Framework was used as a conversation starter for further reflection and discussion on the person’s needs and the extent to which symptoms were affecting the person with dementia. This required the involvement of the family to generate in-depth knowledge of the person with dementia’s needs.

Direct quotes

…because I know both families that I'm using it with, so we've got that rapport already, so I think that helped. – **practitioner**

It wasn't done as a blind sort of survey-type questionnaire, it was done together, both going through it basically – **family carer**

Erm... I suppose it just makes me more aware of how these things are affecting them on a day-to-day basis, and I suppose then it's just increasing or decreasing our support and looking at other things that might be helpful for them. - **practitioner**

It's useful when the clinical staff are here because it opened up a discussion if you like, if there are any sort of symptoms or whatever that need to be discussed, whereas if you're doing it on your own you maybe don't see answers to some of the questions that it actually, that it raises. – **family carer**

Well, it concentrated your mind on what the issues actually were and because you, you know, you're experiencing it full-time all the time you tend to not see things as clearly and it pointed you in the right direction. – **family carer**

The EMBED-Care Framework encouraged active engagement between the family carer to prioritise symptoms and to be involved in care and treatment decision-making. The component of the Framework used here was the clinical decision support tool which the family carer and the practitioner reviewed together after completing the IPOS-Dem. The clinical decision support tool provided guidance for the discussion relating to care and treatment options and offered solutions to the needs of the person with dementia. The family carer valued reviewing the clinical decision support tools together with the practitioner which in turn significantly influenced care and treatment decisions.

Direct quotes

It weren't really until afterwards you're looking at the [clinical decision-support tools] support tool and they made one or two notes of things to actually try and incorporate with what we do day-to-day you know, when there's no one here, it's useful in that respect. – **family carer**

It was really useful to be honest because that's one thing that in normal time you don't do that, you're just reacting to issues as and when but by using that [clinical decision-support tools] tool it gives you guidance if you like on which way and how to approach and prioritise what's supposed to be done. – **family carer**

We kind of looked at the ones that were flagged up red for a start and worked our way down from there. So any that were highlighted red were the ones we kind of focused on to start, so... – **practitioner**

This was important for the family carer to feel informed about care and treatment options. If the person with dementia was able to be involved and a disagreement occurred between the person with dementia and the family carer on priority symptoms for care and treatment, or in agreeing treatment and care plan, the family carer felt that the expertise of the practitioner would be valued in informing the next steps.

Direct quotes

Well basically to advise on what they the next steps are if it is pain management- **family carer**

but you know, I'd speak to them because I see it every day, it's not a case of when someone’s here - **family carer**

A significant challenge with the use of the intervention for the practitioner was the time required to become familiar with the intervention and the time required to use it together with the family carer. The practitioner reported that the use of the EMBED-Care Framework with the family carer took longer than the allocated visit time, but remained positive that the time requirement would reduce with subsequent visits as there is a learning period expected with any intervention.

Direct quotes

Erm... it all depends, to be fair, because some people we can go in and visit for half an hour and that's them done, that's, they're quite happy, they've come, you've gone in, said what you've got to say, they've said what they've got to say and waved you out the door kind of thing, but we're actually allocated about forty five minutes per visit – **practitioner**

but I think that'll just come with time, as we use it more we'll just get used to picking out the relevant bits that people can do something about – **practitioner**

Yeah, so in the first one, where it was just the carer [family carer], it took me just under an hour – **practitioner**

A major concern for the practitioner was patient anonymity protection whilst using the digital version of the intervention. When using the intervention together, the practitioner was required to be sensitive to how they navigated the EMBED-Care Framework app to not reveal other patient information to the family carer.

Direct quotes

Yeah, I think with the laptop it's, you're kind of having to almost hide the other names that are on there when you, because you have to go back on there to actually do the IPOS, don’t you? So you're kind of almost covering the names as you do it or turning the laptop, remembering to turn the laptop round back to you. – **practitioner**

So that's my, that's my biggest thing at the minute, that's really my only issue, that you have to kind of be really mindful, if you're sat with somebody, not to show them the list of people that you're managing [referring to other patients]. – **practitioner**

Yeah, so like if you had them, whether like, I suppose it's even with the plus and minus buttons, so you expand the whole list or keep it not expanded, if that makes sense. – **practitioner**

The family carer and the practitioner highlighted the necessity of flexibility, such as being able to use it before or during the practitioner. The use of the intervention prior to the practitioner visit could enable the family carer to prepare for conversations about the needs of the person with dementia.

Direct quotes

I don't mind either way, it didn't really worry me at all. **– family carer**

Yeah, I think it probably would have helped both of them [referring to both families] to have that erm... like the little bits to have a look through with the symptoms… **- practitioner**

**Objective 2: The feasibility (adherence) and acceptability of the EMBED-Care Framework to enhance shared decision-making.**

In this case, the intervention was used a month after the person with dementia and the family carer were recruited into the study. Once the intervention was set up for this case, it was used at T1 (two weeks apart) and once at T2. All the IPOS-Dem items were completed on all three occasions with no missing items (see figures below). There were no incomplete items on the IPOS-Dem when used. The ‘cannot assess” option was not used.

The family carer perceived the intervention as a whole to be “easy” to use and liked the digital app because of the faster use over paper.

Direct quotes

No, erm no, it was fairly easy to go through which makes a big difference I think, it wasn't that complicated at all, it was fairly straightforward. **- family carer**

It's sort of quicker and easier. **- family carer**

Yeah, I don't mind either to be perfectly honest but you know, it's easier going through the app than it is a bunch of paperwork I think these days. **- family carer**

**Case 2 IPOS-Dem radar plots**

|  | T1 - first use | T1 - second use | T2 |
| --- | --- | --- | --- |
| Diarrhoea | 0 | 0 | 0 |
| Hallucinations | 0 | 1 | 1 |
| Agitation | 1 | 2 | 1 |
| Wandering | 0 | 1 | 2 |
| Feeling anxious or worried? | 3 | 1 | 1 |
| Family's anxiety and worry | 3 | 2 | 1 |
| Have you been feeling depressed? | 1 | 0 | 0 |
| Loss of interest in usual activities | 2 | 1 | 2 |
| Feeling at peace | 2 | 3 | 2 |
| Positive interaction with others | 1 | 1 | 3 |
| Have practical problems been addressed? [e.g. hearing aids, foot care, glasses, diet] | 0 | 1 | 0 |

|  | T1 - first use | T1 - second use | T2 |
| --- | --- | --- | --- |
| Pain | 2 | 2 | 2 |
| Shortness of breath | 1 | 1 | 0 |
| Weakness or lack of energy | 2 | 2 | 2 |
| Nausea | 0 | 0 | 0 |
| Vomiting (being sick) | 0 | 0 | 0 |
| Poor appetite | 0 | 1 | 1 |
| Constipation | 0 | 2 | 2 |
| Dental problems or problems with dentures | 1 | 1 | 0 |
| Sore or dry mouth | 0 | 1 | 1 |
| Drowsiness (sleepiness) | 3 | 2 | 2 |
| Poor mobility | 2 | 2 | 4 |
| Swallowing problems | 0 | 1 | 2 |
| Skin breakdown | 1 | 2 | 2 |
| Difficulty communicating | 0 | 2 | 2 |

*Note: lower score is interpreted as less severe symptoms

**Case two utility questionnaire table**

The Utility questionnaire showed that the intervention was used together by the family carer and practitioner, indicating that the IPOS-Dem and clinical decision support tools were used was with the family carer.

**The priorities of care list used**

The app data showed that the priorities of care list was used on one occasion across T1 and T2. The priorities of care list table above was the only example of a completed version.

*= The person with dementia lacked capacity to participate in the assessment process and discussion about care and treatment.

Case three summary

**Demographics**

**Person with dementia**

Clinical Dementia Rating Scale: 1 (mild dementia)

Case 3 Edmonton Symptom Assessment Scale score: 45 (out of 100), although four symptoms received a score of five or more. These were pain, tiredness, drowsiness, shortness of breath and anxiety.

| Pain | Tiredness | Drowsiness | Nausea | Lack of appetite | Shortness of Breath | Depression | Anxiety | Wellbeing |
| --- | --- | --- | --- | --- | --- | --- | --- | --- |
| 9 | 7 | 7 | 3 | 3 | 4 | 4 | 6 | 2 |

**Family Carer**

Relationship between the person with dementia and family carer: Mother/daughter (family carer)

**Practitioner**

Role: Nursing Assistant

Number of years of clinical experience: 5-9 years

**Summary of how the case used the intervention**: In this case, participants used the EMBED-Care Framework app. The person with dementia was non-verbal and, therefore, was not involved in using the EMBED-Care Framework; rather, it was used with the family carer. The practitioner, a nursing assistant, used the intervention with the family carer during a home visit appointment. The staff version of the IPOS-Dem was completed. The DSTs supported discussion on care and treatment after completing the IPOS-Dem. The priorities of care list was unused. The support resources were unused.

**Objective one – to explore the processes of how the EMBED-Care Framework enhances shared decision-making for people affected by dementia living at home and practitioners**

The first step in using the EMBED-Care Framework was the role of the practitioner in explaining the intervention and its intention. The practitioner communicated with the family carer about the EMBED-Care Framework and its purpose. The practitioner knew the family carer well; therefore, rapport was already established. The practitioner felt that it was important to involve the person with dementia if they’re in the milder stages of dementia as a way of eliciting information about their symptoms and concerns that they may not share with the family carers. The person with dementia was present but not able to be involved in symptom discussion and decision-making. However, it was critical that family carers be involved in using the EMBED-Care Framework to provide an in-depth insight into the person with dementia’s needs. The practitioner welcomed the opportunity to collaborate with the family carer to reflect and discuss the needs of the person with dementia, using the IPOS-Dem as a tool to facilitate this conversation. The family carer valued the monitoring aspects of the IPOS-Dem to report and track changes over time.

Direct quotes

…because sometimes she does notice different symptoms, and we’re not going for another week or two, and she’ll be worried about them, or she won’t know what actions to take as such. – **practitioner**

So I think it’s really important to get families involved, I think that’s a really good idea, yeah. - **practitioner**

we went through it together, then she had a go by herself, and then asked me if am I doing it right, so I can go through it with her. – **practitioner**

Yes, we went through it together. – **family carer**

So they can see what kind of questions and symptoms, because they may be different to me, I think it’s really useful for them to have a look at it with you, because if they’ve got any questions as well they can ask you any questions. And sometimes if I notice a different symptom and she hasn’t noticed it, we can go through it together and she can speak with me, or ask me any questions if she needs to. - **practitioner**

The clinical decision support tools were reviewed by the practitioner and family carer, informed by the results of the IPOS-Dem assessment. The clinical decision support tool prompted further discussions to identify care and treatment options, but the family carer’s preferences.

Direct quotes

It was pretty good, because it was like, it was in two stages so it would be like you know if mum’s doing this you could try this. If that didn’t work then, you know, there was like who to get in touch with or you know what I mean, it was quite easy to follow, which will be quite good. **– family carer**

No, not really, no. No, it was like more, it was self-explanatory really and it was just like you know, it was quite useful. **– family carer**

So yeah, we learnt something new about [the person with dementia] then, and then we spoke about what kind of foods [the person with dementia] would prefer to eat, just because of the texture, and things like that, and the swallowing. – **practitioner**

In this case, the person with dementia was not able to be involved, however the family carer reflected on how they would wish multiple priorities and preferences to be managed if the person with dementia was able to be involved and had differing priorities to the family carers. The family carer expressed that they would discuss with the practitioner.

Direct quotes

I think, in that case I think, you know, I would probably speak to [practitioner] and just like you know discuss it that way – **family carer**

The family carer highlighted the efficiency of the digital app as a key point for using the EMEBD-Care Framework. Flexibility was important to the family carer in terms of when and how the EMBED-Care Framework is used, e.g. together with the practitioner or alone.

Direct quotes

Yes, so I let her know that I’ll get notified every 28 days, to complete a new one, because [the person with dementia] does sometimes have a few new symptoms, sometimes it could be weekly or fortnightly, so I said if she notices anything, she can pop IPOS-Dem on there **– practitioner**

I think I would do it by myself as well, so you know like I say, I know that it gives you like notifications if you put anything, added anything to it. And when I’m set up on it with my password, then if [practitioner?] puts anything down it will flag up on my phone or if I put anything down it’ll flag up for them. So, that’s pretty good. - **family carer**

No, I think, I mean [the practitioner] did say that like when I do first start it, she will be with me to go through it in case I got stuck, but you know I think I would have a good attempt at doing it. - **family carer**

**Objective 2: The feasibility (adherence) and acceptability of the EMBED-Care Framework to enhance shared decision-making.**

The app data showed that the intervention, specifically the IPOS-Dem and clinical decision support tools, were not used at T1 but were used at T2. The priorities of care list was not used. The research field notes suggested that there was an initial lack of confidence to use the intervention. However, once used, the family carer and the practitioner valued using the EMBED-Care Framework together and had a positive attitude towards it, reporting that it is a “good set-up” and “And I think the families all feel a lot more involved with us, as well, which is nice…” - practitioner. There were no incomplete items on the IPOS-Dem when used. The ‘cannot assess” option was not used.

The qualitative data for this case showed that the shared decision-making intervention was acceptable, with the participants referring to its use as “straightforward” and “easy”, despite feeling that they required more time to become familiar with the whole intervention.

Direct quotes

I think it’s easy enough to, if they’re at the beginning stages I’d say, but I think after a certain stage of the dementia, I don’t think they’d be able to use it, not because of the app, but just because of their dementia. – **practitioner**

So that really did help and when you’ve done it a few times it kind of comes naturally, because it’s that easy to use when you’ve done it a few times. – **practitioner**

Yeah, I think, like I say you know once I get into it, I think it’s like with everything, once you get into it and you know where to look for things it’s easy. – **family carer**

I think the app. I have that much paperwork with work, I think the app would be, you know, sufficient. – **family carer**

I think because it is on the phone and like I say if there is any notifications from [practitioner] I can pick them up straight away. Or you know, if I put anything then [practitioner] can pick them up straight away. – **family carer**

**Radar plot of the IPOS-Dem items over time**

|  | T1 |
| --- | --- |
| Sleeping problems | 0 |
| Diarrhoea | 1 |
| Hallucinations | 0 |
| Agitation | 3 |
| Wandering | 0 |
| Feeling anxious or worried | 2 |
| Family’s anxiety and worry | 4 |
| Feeling depressed | 3 |
| Loss of interest in usual activities | 3 |
| Feeling at peace | 2 |
| Positive interactions with others | 1 |
| Have practical problems been addressed? | 0 |

|  | T1 |
| --- | --- |
| Pain | 1 |
| Shortness of breath | 0 |
| Weakness or lack of energy | 1 |
| Nausea | 0 |
| Vomiting | 0 |
| Poor appetite | 2 |
| Constipation | 0 |
| Dental problems | 1 |
| Sore or dry mouth | 1 |
| Drowsiness (sleepiness) | 0 |
| Poor mobility | 1 |
| Swallowing problems | 3 |
| Skin breakdown | 0 |
| Difficulty communicating | 4 |

**Utility questionnaire responses of how the intervention was used**
